# Alterations of HIV-1 envelope phenotype and antibody-mediated neutralization by signal peptide mutations

**DOI:** 10.1371/journal.ppat.1006812

**Published:** 2018-01-25

**Authors:** Chitra Upadhyay, Roya Feyznezhad, Weiming Yang, Hui Zhang, Susan Zolla-Pazner, Catarina E. Hioe

**Affiliations:** 1 Icahn School of Medicine at Mount Sinai, Division of Infectious Diseases, New York, New York, United States of America; 2 Department of Pathology, Johns Hopkins University, Baltimore, Maryland, United States of America; 3 James J. Peters Veterans Affairs Medical Center, Research Service, Bronx, New York, United States of America; University of Zurich, SWITZERLAND

## Abstract

HIV-1 envelope glycoprotein (Env) mediates virus attachment and entry into the host cells. Like other membrane-bound and secreted proteins, HIV-1 Env contains at its N terminus a signal peptide (SP) that directs the nascent Env to the endoplasmic reticulum (ER) where Env synthesis and post-translational modifications take place. SP is cleaved during Env biosynthesis but potentially influences the phenotypic traits of the Env protein. The Env SP sequences of HIV-1 isolates display high sequence variability, and the significance of such variability is unclear. We postulate that changes in the Env SP influence Env transport through the ER-Golgi secretory pathway and Env folding and/or glycosylation that impact on Env incorporation into virions, receptor binding and antibody recognition. We first evaluated the consequences of mutating the charged residues in the Env SP in the context of infectious molecular clone HIV-1 REJO.c/2864. Results show that three different mutations affecting histidine at position 12 affected Env incorporation into virions that correlated with reduction of virus infectivity and DC-SIGN-mediated virus capture and transmission. Mutations at positions 8, 12, and 15 also rendered the virus more resistant to neutralization by monoclonal antibodies against the Env V1V2 region. These mutations affected the oligosaccharide composition of N-glycans as shown by changes in Env reactivity with specific lectins and by mass spectrometry. Increased neutralization resistance and N-glycan composition changes were also observed when analogous mutations were introduced to another HIV-1 strain, JRFL. To the best of our knowledge, this is the first study showing that certain residues in the HIV-1 Env SP can affect virus neutralization sensitivity by modulating oligosaccharide moieties on the Env N-glycans. The HIV-1 Env SP sequences thus may be under selective pressure to balance virus infectiousness with virus resistance to the host antibody responses. (289 words)

## Author summary

HIV-1 envelope glycoprotein (Env) is indispensable for virus infection. HIV-1 Env contains at its N terminus a signal peptide (SP) that directs the protein to the endoplasmic reticulum. The SP sequences exhibits high variability among HIV-1 isolates, and the significance of such variability is unclear. We hypothesize that changes in the Env SP influence the Env biogenesis, Env folding and/or glycosylation and the phenotypic traits of the virus. This study evaluated the consequences of mutations in the Env SP of infectious molecular clone HIV-1 REJO.c/2864. Results show that three different mutations affecting histidine at position 12 impacted on the Env incorporation into virions that correlated with virus infectivity and transmission. Additionally, Env SP mutations at positions 8, 12, and 15 increased virus resistance to neutralization by Env monoclonal antibodies. These mutations also altered the oligosaccharide composition of N-glycans on Env as shown by changes in the Env reactivity with lectins and by mass spectrometry. Similar phenotypic changes were observed when analogous SP mutations were introduced to another virus strain, JRFL. Thus, the HIV-1 Env SP controls Env expression and glycosylation that affect virus infectivity, transmission, and sensitivity to neutralization by antibodies. (191 words)

## Introduction

The HIV-1 envelope glycoprotein (Env) is synthesized as a gp160 precursor protein that is cleaved into gp120 (the receptor-binding subunit) and gp41 (the transmembrane subunit). Three non-covalently linked gp120-gp41 heterodimers assemble to form a functional trimeric Env spike expressed on the virion surface. All membrane-bound and secreted proteins, including HIV-1 Env, contain N-terminal signal peptides (SP) that target the nascent polypeptides to the endoplasmic reticulum (ER). During the transport through the ER and subsequently the Golgi apparatus, HIV-1 Env is subjected to extensive glycosylation that adorns ~30 potential N-linked glycans on each gp160 molecule. Disulfide bonds are also formed, enabling the protein to adopt the appropriate conformation and oligomerization. Although SP is clearly a critical element that determines the glycosylation, folding, and trimerization of HIV-1 Env, very little is known about the contribution of its amino acid composition.

**Fig 1:**
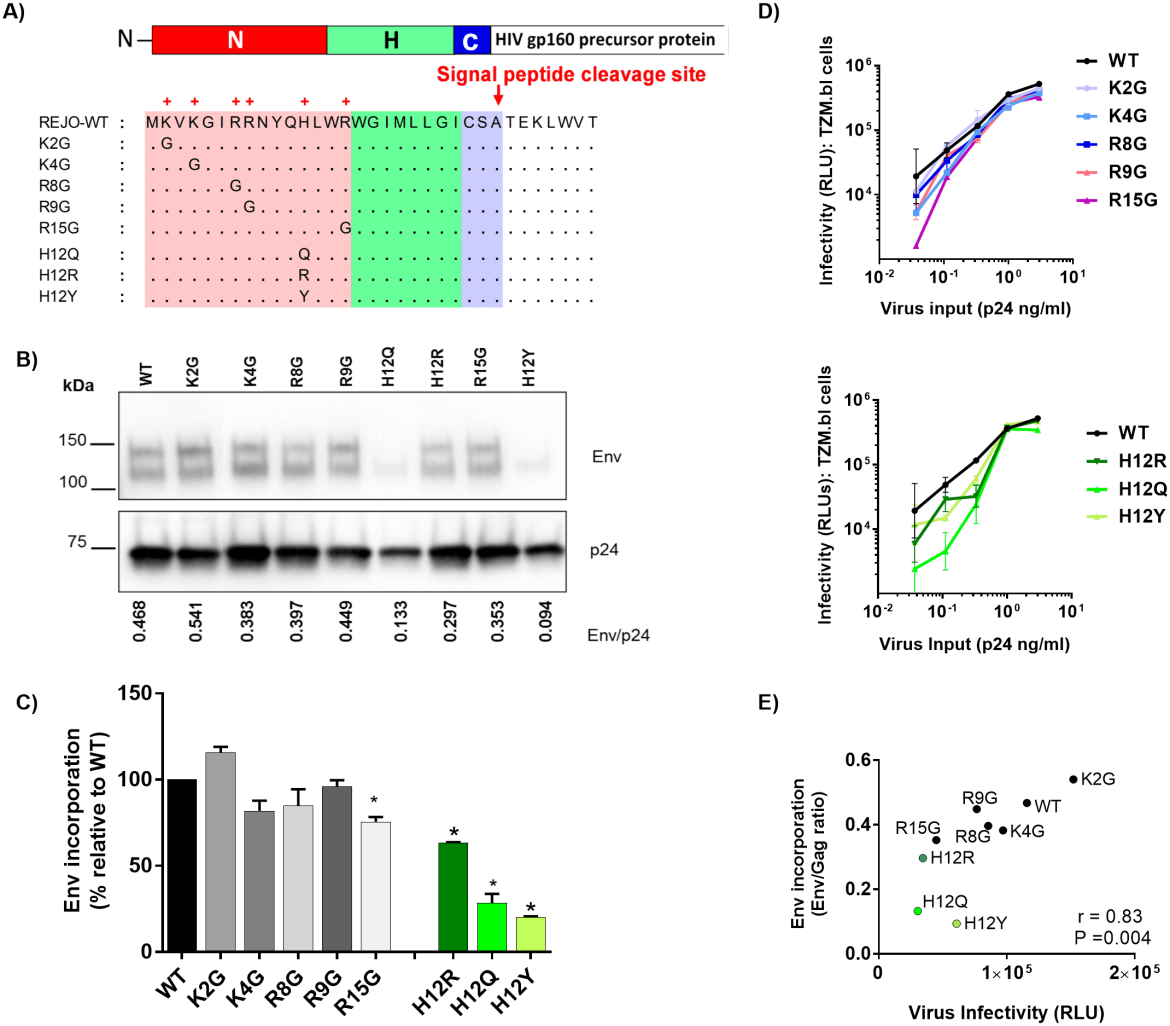
Effects of SP mutations on the REJO Env expression and virus infectivity. (A) Schematic representation of REJO WT and eight different SP mutations evaluated in this study. (B) Measurement of Env incorporation by Western blot. WT and mutant viruses were produced in transfected 293T cells, lysed, and analyzed by SDS-PAGE (4–20%) and Western blot. An anti-gp120 MAb cocktail (V3: 391/95-D, 694/98-D, 2219, 2558; C2: 847-D, 1006-30D; C5: 450-D, 670-D) and a p24 Gag MAb (91–5) were used to detect the relative levels of Env and Gag associated with virions. The ratios of Env/Gag were calculated. (C) The levels of Env incorporation into the mutant virions relative to that of WT were calculated based on their Env/Gag ratios (WT value was normalized to 100%). *, p< 0.01 (ANOVA). (D) Infectivity of WT vs. mutant viruses (K/R: top; H12: bottom) in CD4+ TZM.bl cells exposed to titrated viruses with equivalent p24 contents. (E) Correlation of virus infectivity in CD4+ TZM.bl cells with the incorporation of Env into virions by Spearman’s rank test. Infectivity was based on RLU produced upon infection with a fix amount of virus input (0.33 ng p24/ml).

Like other SPs, the HIV-1 Env SP consists of three segments: a cationic N-terminus, a central hydrophobic region and a C-terminal region. However, SPs are highly diverse in terms of the lengths and the amino acid sequences. For examples, the SP of the vesicular stomatitis virus G protein (VSV-G) is only 16 amino acids long [1], while the SPs of the Env proteins from feline immunodeficiency virus and foamy virus are 87 and 187 amino acids long respectively [2, 3]. The SP of HIV-1 Env consists of about 30 amino acids with a ~15 amino acids long N-terminus bearing charged residues, a hydrophobic region of ~11 amino acids essential for the translocation of the newly synthesized polypeptide chain to the ER membrane, and a ~3 amino acids long C-terminal region that contains the cleavage site for the signal peptidase [4] (Fig 1A). Among SPs of proteins in general, the highest diversity is found in the N-terminal region, and the charged residues in this region influence the ER translocation function of the neighboring hydrophobic region [5]. The SP of HIV-1 Env is unique; it has an unusually long N-region with a relatively high number of positively charged amino acids [6]. The HIV-1 Env SP is also cleaved post-translationally as opposed to the SPs of many other proteins which are cleaved co-translationally [6–8]. Hence, the HIV-1 gp160 remains N-terminally attached to its SP for some time after the completion of its synthesis [7]. Nonetheless, SP cleavage is required for Env secretion; Env with uncleaved SP is retained in the ER and degraded [9]. It is hypothesized that the SP regulates the HIV-1 Env biogenesis [6–8] not only by controlling the timing of Env binding to the ER chaperones but also by influencing the HIV-1 Env folding and glycosylation [8, 10]. Furthermore, in view of the fact that a high degree of variability is found among the Env SPs from different HIV-1 isolates, the SP sequence variation is likely to modulate the Env structure and glycosylation to impact on virus interactions with cells and immune system of the host.

Comparison of the HIV-1 Env SPs from the different strains and clades reveals remarkable amino acid variability (S1 Fig). The study by da Silva et al. [11] also reported deletions of neutral and basic residues at the amino terminal Env SP in viruses from early stages and insertion of basic residues in the hydrophobic region in late-stage isolates. Comparison of Env between pairs of newly infected individuals and their transmitting partners similarly showed mutations in Env SP [12, 13]. However, the significance of such variations is yet unclear. Using a computational strategy, Gnanakaran et al. [14] identified several signature amino acids in the HIV-1 Env glycoprotein. Of particular interest was the loss of histidine at position 12 in the Env SP that was associated with the transition from acute to chronic viruses [14, 15]. Gonzalez et al. also reported a similar signature motif in the Env SPs of SIV [16]. The amino acid changes accumulated in the SP during virus evolution from the acute stage to the chronic stage of infection implicate a potential role of the SP in immune evasion, presumably by controlling the differential Env expression levels, folding, or post-translational modifications. Consistent with this idea, swapping the gp120 SP with a heterologous SP or decreasing the number of positively charged amino acids were found to increase gp120 expression and secretion (7–11). However, these past studies were done mainly in the context of recombinant Env proteins and the effects of the Env SP variability on the virus have not been much studied.

This study sought to evaluate the importance of the amino acid polymorphism at position 12 and the contribution of charged amino acids in the N-terminal region of the HIV-1 Env SP on the functions of Env expressed by full-length infectious molecular clones (IMCs). The data show that some Env SP mutations altered the level of Env incorporated into virions without drastic effect on virus infectivity. Certain mutations also rendered the virus resistant to neutralization by anti-V1V2 antibodies. Similar changes in neutralization sensitivity were observed when analogous mutations were introduced in the Env SPs of two different HIV-1 isolates (REJO and JRFL). We postulate that the Env SP mutations impact Env glycosylation which in turn masks or exposes the neutralizing epitopes. Lectin-probed Western blot and mass spectrometric analyses demonstrate that indeed these mutations affected the sugar composition of N-glycans that decorate the HIV-1 Env. Thus, the data support the hypothesis that the amino acid variability in HIV-1 Env SP shapes Env glycosylation to affect Env recognition by antibodies. These findings highlight the importance of SP in regulating the N-glycan composition of HIV-1 Env.

## Results

### Effects of mutations in the HIV-1 REJO Env SP on Env incorporation and virus infectivity

We began our exploration to study the effects of mutations in the Env SP by examining the relative levels of Env and Gag incorporated into virions. 293T cells were transfected with the REJO WT and mutant constructs shown in Fig 1A. Forty-eight hrs later viruses released into the supernatants were pelleted and analyzed by Western blot for Env and Gag levels (Fig 1B). We calculated the ratios of Env/Gag to compare Env incorporation among the WT and mutant viruses. The SP mutations altered the ratios of Env/Gag to varying extents. The ratios ranged from 115% to 20% in comparison to WT (set to 100%), (Fig 1C). Interestingly, three mutations affecting H12 residue (H12Q, H12R, and H12Y) caused the most reduction in Env incorporation. The mutations affecting basic K or R residues at positions 4, 8, 9, and 15 minimally reduced the ratios of Env/Gag incorporated into virions, while in K2G mutant Env was incorporated into virions at a slightly higher density compared to the WT. The levels of Env expression in the cell lysates were also lower for R15G and H12 mutants (H12Q, H12R, and H12Y) (S2A and S2B Fig), similar to those found in the virions, whereas higher amounts of K2G and R9G Env were detected in the cell lysates. Of note, two Env bands (120kDA and 140kDa), which reacted with anti-gp120 MAbs, were observed in both virus and cell lysates (Fig 1B and S2 Fig). Similar bands were also seen in virus preparation purified using sucrose cushion. The blots were then probed with anti-gp41 MAbs: A cocktail of 7 anti-gp41-specific MAbs did not react with either bands, although it strongly detected gp41 in the same samples (S3A Fig). In contrast, the anti-gp41 MAb 2F5, specific to the MPER region, weakly recognized the upper and not the lower Env band (S3B Fig), suggesting that these two Env bands are cleaved gp120 and uncleaved gp160. The 2F5 also detected the gp41.

Subsequently, the relative rate of Env production was measured in cells after transfection using a sandwich ELISA with capturing antibody specific for the C-terminus of gp120 (C5) and probing with MAb EH21 against C1 epitope at the gp120 N-terminus or MAb A32 recognizing a conformational-dependent epitope involving C1, C2, and C4. This assay detects properly-folded gp120 Env with accessible N- and C-termini that result from cleavage of both signal sequence and gp41. The data demonstrate comparable rates of gp120 production with WT vs mutated SP (S2C Fig), indicating that the SP mutations did not drastically alter Env synthesis and proteolytic cleavage.

The infectivity of REJO WT and mutant viruses was evaluated in TZM.bl cells at 48 hours after one round of infection with virus inputs normalized by p24 contents. None of the mutations completely abrogated virus infection. Among mutations affecting basic residues at positions 2, 4, 8, 9, and 15, the R15G mutation reduced virus infectivity the most, while the others had minimal effects (Fig 1D). The three H12 mutations also lowered virus infectivity, albeit to different extents and evident only at lower p24 concentrations. A significant correlation (Spearman *r* = 0.83, *P* = 0.004) was observed between virus infectivity and virus-associated Env/Gag ratio (Fig 1E), indicating that virus infectivity is impacted by reduced Env incorporation into virions as a result of SP mutations.

### Effects of SP mutations on REJO Env gp120 binding by antibodies

To evaluate whether the SP mutations alter the Env antigenicity, solubilized gp120 proteins from REJO WT and mutant viruses were tested in ELISA with MAbs specific for V2i (697, 1357, 1361, 1393 and 2158), V3 (2219, 2557, 3074 and 3869) [17], the CD4-binding site (CD4bs: NIH45-46) [18], and a CD4-IgG fusion protein (CD4-IgG2, Progenics). The V2i MAbs target distinct epitopes that are nearby or overlap with the integrin α4β7 binding site in the V1V2 domain. MAb 1418 specific for human parvovirus B19 was included as a negative control [19]. The same amounts of HIV-1 Env gp120 (100 μl/well at 20 ng/ml) from the different mutant and WT viruses were added to ELISA wells pre-coated with capturing anti-C5 antibody. The S4 Fig show that, except for H12Q, the SP mutations minimally affected Env gp120 recognition by the MAb panel. None of the mutations completely abolished MAb binding with gp120, although the V2i MAb 1357 reacted weakly with WT and the SP mutants. Notably, the H12Q mutation caused the greatest reduction of Ab binding: the binding of H12Q gp120 by all five V2i MAbs, two V3 MAbs (3074 and 3869), and the CD4bs MAb NIH45-46 decreased by >25% relative to WT. However, H12Q did not affect the binding of two other V3 MAbs (2219 and 2557). The other mutations only sporadically lowered the strength of gp120-MAb reactivity. In contrast, all mutants interacted less efficiently with CD4-IgG2. These data revealed structural changes that may be promulgated from SP to affect the MAb epitopes and the CD4 binding site in the Env gp120 subunit.

### Effects of SP mutations on the sensitivity of HIV-1 REJO to neutralization by antibodies

**Fig 2:**
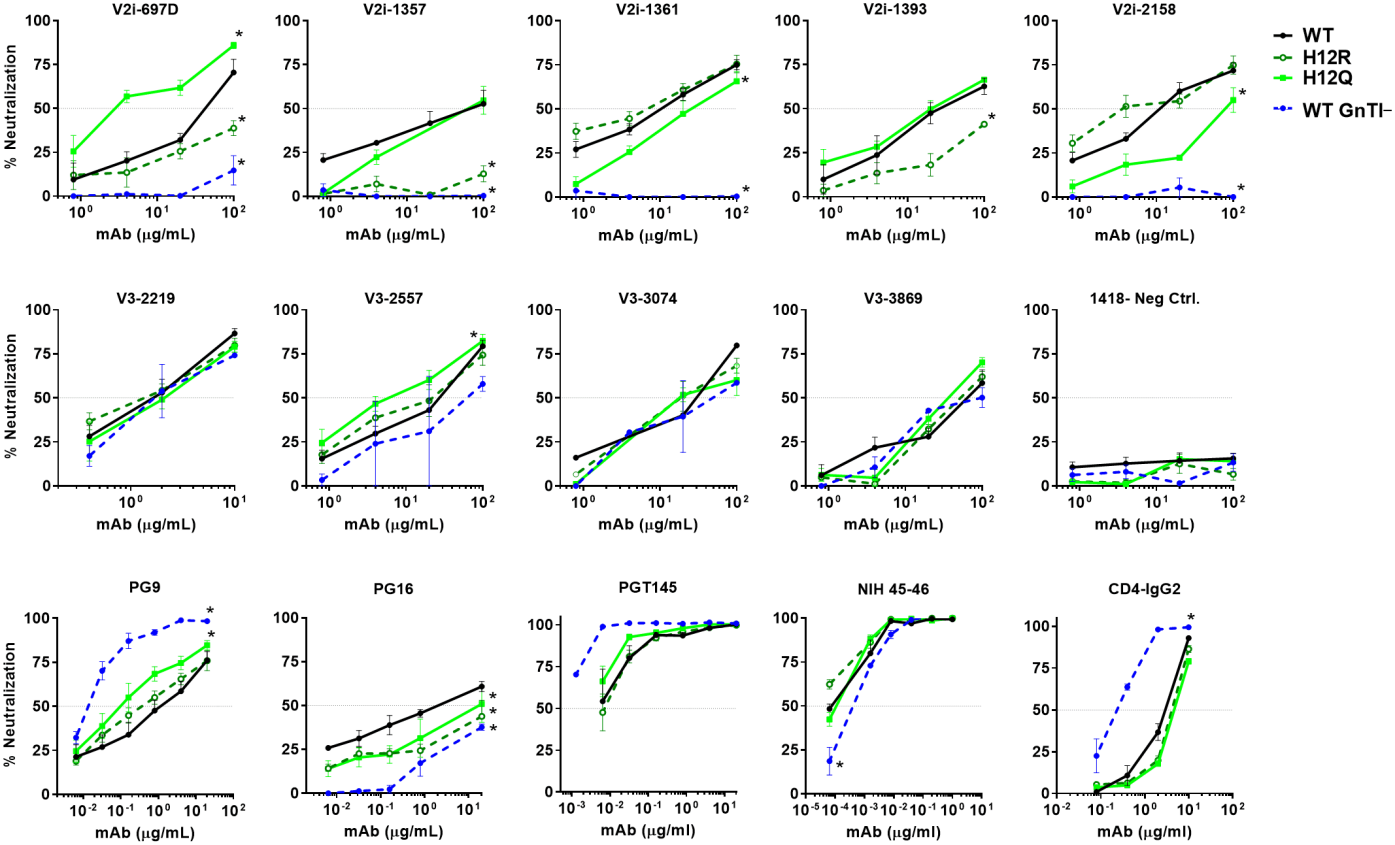
Neutralization of REJO WT and mutant viruses by different MAbs and CD4-IgG2. Virus neutralization was measured using the TZM.bl target cells after the viruses were incubated with serially diluted MAbs at 37°C for 24 hours prior to addition of the target cells, except for PG9, PGT145, NIH45-46, and CD4-IgG2 which were pre-incubated with viruses for 1 hour. The irrelevant control MAb 1418 was included for negative control. Means and standard errors from two to three independent experiments are shown. *, p<0.05 from the two-way ANOVA as compared to neutralization of the WT virus. Data from two SP mutants (H12R and H12Q) are shown. Neutralization data of WT REJO produced in 293S GnTI^-^ cells (WT GnTI^-^) are also presented for comparison.

**Fig 3:**
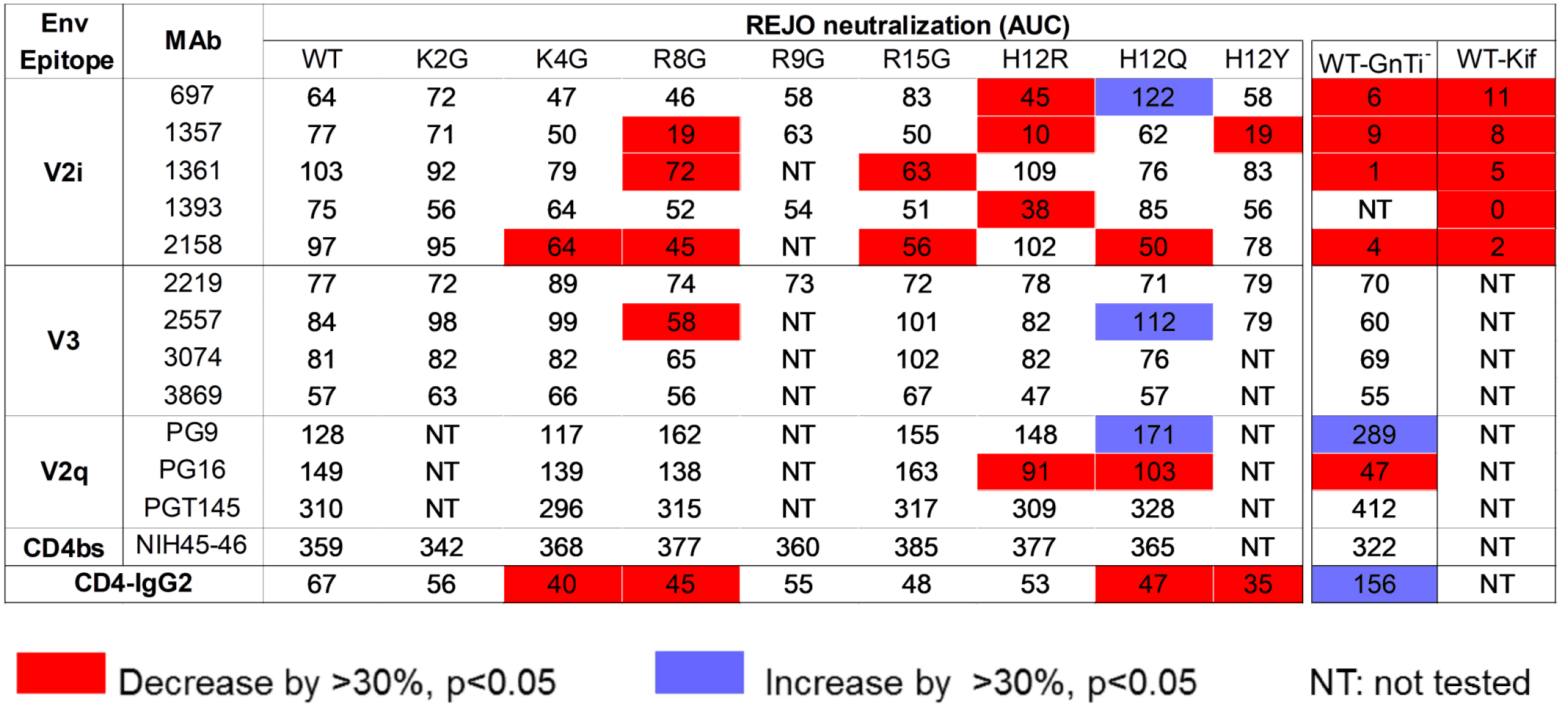
AUC values of REJO WT and SP mutant neutralization by MAbs targeting V2i, V3, V2q, and the CD4bs and by CD-IgG2. Neutralization assays were performed for each of the MAb-virus pairs as described in Fig 2, and areas under the titration curves (AUCs) were calculated. AUCs that decreased or increased by >30% and had p<0.05 relative to the corresponding values for WT neutralization by the same MAbs or CD4-IgG2 are shown in red or blue. Neutralization data of REJO WT viruses produced in 293S GnTI^-^ cells or in 293T cells in the presence of kifunensine are included for comparison.

**Fig 4:**
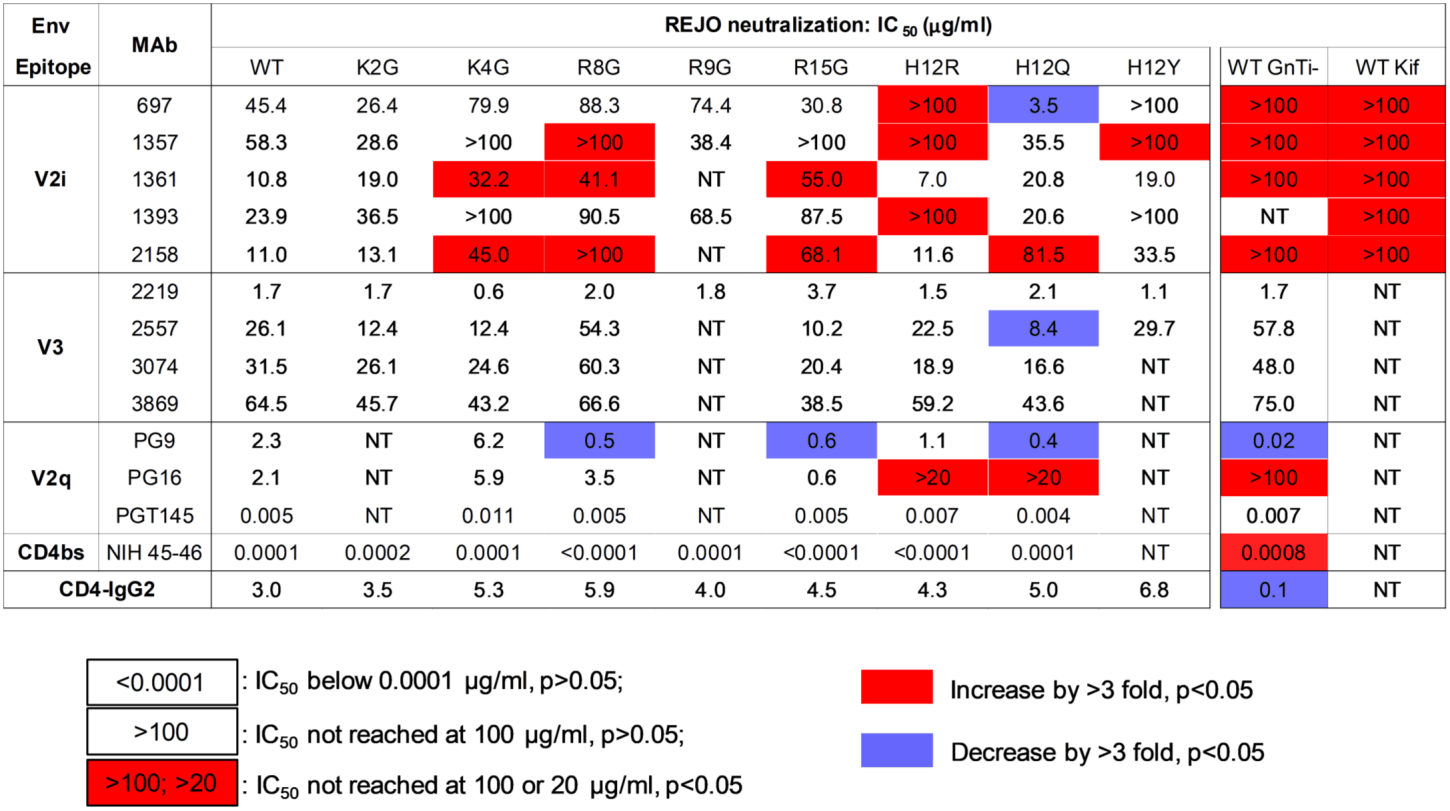
IC_50_ values of REJO SP mutants vs. WT by MAbs targeting V2i, V3, V2q, and the CD4bs and by CD-IgG2. Neutralization assays were described in Fig 2. IC_50_ (μg/ml) values that increase or decrease by >3 fold and p<0.05 as compared to that of WT by the same MAbs or CD4-IgG2 are shown in red and blue. Data from REJO WT viruses produced in 293S GnTI- cells or in 293T cells in the presence of kifunensine are also presented.

Although most of the SP mutations only minimally altered MAb reactivity with soluble gp120 monomers, we postulated that they might cause more dramatic changes to virus neutralization sensitivity as a result of altered assembly and/or glycosylation of the native Env trimers on HIV-1 virions. To test this idea, we examined neutralization of REJO WT and mutants by a panel of V2i, V3, and CD4bs MAbs used in ELISA plus bNAbs specific for the quaternary V1V2 (V2q) epitopes (PG9, PG16, PGT145) and CD4-IgG2. Our past work demonstrated that increasing the virus-MAb incubation time to 24 hours before addition of TZM.bl target cells allowed the detection of neutralizing activities by V2i and V3 MAbs against the Tier 2 viruses REJO and JRFL, while no neutralization was detected with these MAbs with the standard 1-hour pre-incubation [17]. Hence, we assessed REJO neutralization with 24 hours of virus-MAb pre-incubation for all MAbs, except for bNAbs (V2q MAbs: PG9, PGT145; CD4bs MAb: NIH45-46) and CD4-IgG2. Virus input was set to 150,000 to 100,000 RLU in TZM.bl cells. AUC and IC_50_ values were calculated from titration curves. Titration curves from two of the mutants, H12R and H12Q, are shown in Fig 2, whereas AUC and IC_50_ data from all virus and MAb combinations were tabulated in Figs 3 and 4.

The data showed that the SP mutants displayed varied sensitivity to different MAbs (Figs 2, 3 and 4). Notably, many mutations rendered REJO resistant to neutralization by V2i MAbs. In contrast, neutralization by V3 MAbs and the bNAbs against V2q and CD4bs was less affected. The H12R mutant, for example, was more resistant than WT to V2i MAbs 697D, 1357 and 1393. This mutant also displayed increased resistance to the V2q MAb PG16, which recognizes a glycopeptidic epitope and interacts specifically with sialic acid on the complex-type glycans [20]. On the other hand, H12R neutralization by all V3 MAbs, PGT145, NIH45-46, and CD4-IgG2 was comparable to WT. Similar patterns were observed with K4G, R8G, R15G, and H12Y (Figs 3 and 4), although K4G, R15G, and H12Y each affected only 1 or 2 V2i MAbs. In contrast, the H12Q mutant became more sensitive than WT to V2i MAb 697, V3 MAb 2557, and V2q MAb PG9. The changes were not drastic for MAbs 2557 and PG9, but the shifts in the titration curves were consistently observed that affected both AUC and IC_50_ values. Nonetheless, not all SP mutations altered neutralization sensitivity. K2G mutation did not affect REJO neutralization sensitivity to any V2i or V3 MAbs tested. R8G and R15G mutants showed higher sensitivity to PG9 as compared to WT; however, although their IC50 values differed by >3 fold (p<0.05), their AUC values had only 28–29% difference. Effects on the V3 glycan-specific MAbs such as PGT121 and PGT128 and the mannose-binding MAb 2G12 could not be assessed, because REJO Env does not have the N322 glycan essential for recognition by this class of MAbs. Overall, based on AUC (Fig 3), 11 of 38 (29%) REJO mutant-V2i MAb combinations tested became more resistant, while only 1 combination of 27 (4%) showed more resistance to V3 mAb.

Altered patterns of virus neutralization were similarly observed with WT REJO produced in HEK293S GnTI^-^ (GnTI^-^) or in HEK293T cells with kifunensine. The GnTI^-^ cells are deficient in N-acetylglucosaminyltransferase I, an enzyme required for Man_5_GlcNAc_2_ progression into hybrid and complex carbohydrates in the Golgi. Thus, virus produced in GnTi^-^ cells has an increased amount of Man_5-9_ GlcNAc_2_ [21]. On the other hand, kifunensine is a glycosidase inhibitor that prevents the trimming of Man_9_GlcNAc_2_ by the ER mannosidase I enzyme. Virus grown in the presence of this inhibitor displays mainly Man_8-9_GlcNAc_2_ at the utilized N-linked glycosylation sites. Figs 2, 3 and 4 show that REJO WT produced in GnTI^-^ cells (WT GnTI^-^) or grown with kifunensine (WT Kif) were resistant to V2i MAbs, mimicking the phenotypes of many SP mutants. Neutralization by V3 MAbs, on the other hand, was not altered. As expected, neutralization by PG9 of which recognition depends on high-mannose glycans was enhanced, whereas neutralization by PG16 which binds to complex-type glycans was reduced [20]. Neutralization of WT GnTI^-^ virus by the CD4bs MAb NIH45-46 and CD4-IgG2 was also altered, albeit in opposing ways.

### Effects of SP mutations on REJO virus capture and transmission by DC-SIGN

**Fig 5:**
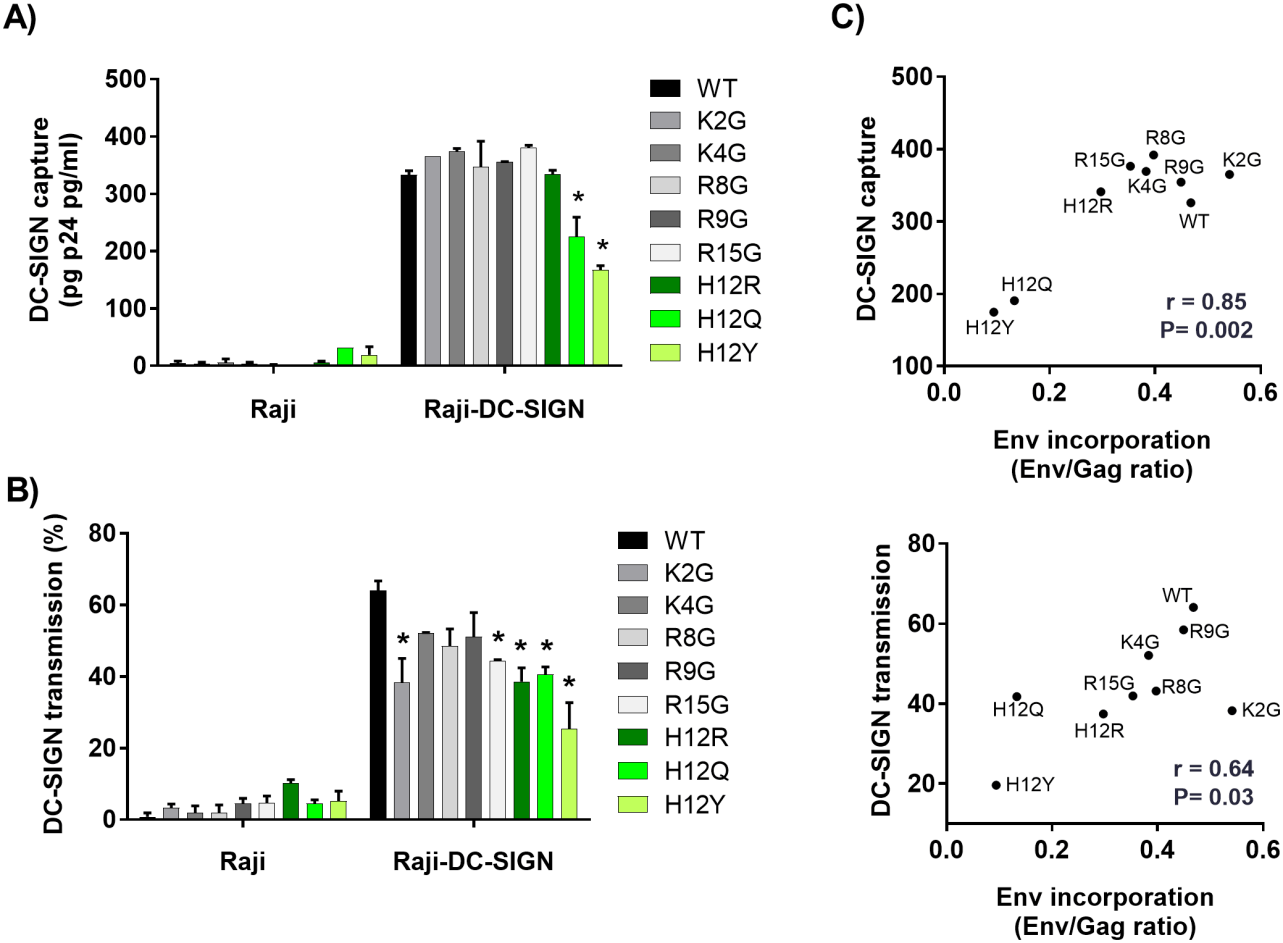
Effects of SP mutations on REJO virus capture and transmission by DC-SIGN. (A) Parental Raji or Raji–DC-SIGN^+^ cells were incubated for 2 hours with WT or mutant viruses produced in 293T cells. Cells were washed extensively, and the amounts of p24 protein associated with the cells were measured by ELISA. (B) Parental Raji or Raji–DC-SIGN^+^ cells were incubated with WT and mutant viruses for 2 hours, washed to remove unbound viruses, and added to CD4+ TZM.bl cells. Viral transmission to the TZM-bl cells was determined by luciferase activity and calculated based on infection in TZM.bl cells without Raji cells as control (set to 100%). Background luciferase activity was determined in co-cultures without any virus. *, p< 0.05 as compared to WT (ANOVA). (C) Correlation of virus capture (top) and transmission (bottom) via DC-SIGN with the Env incorporation into the WT and mutant virions by Spearman’s rank test.

Because SP mutations conferred significant alterations in HIV-1 sensitivity to neutralizing MAbs that may be associated with Env glycan modifications, we next examined whether the mutations also affect the ability of DC-SIGN to bind and transmit HIV-1. DC-SIGN is a C-type lectin which recognizes selective arrays of high mannose- and complex-type N-glycans [22]. Expressed on dendritic cells, DC-SIGN participates in mediating HIV-1 capture and transfer from dendritic cells to T cells [23]. The relative efficiencies of DC-SIGN-mediated capture of REJO WT and mutants were assessed using DC-SIGN+ Raji cells and measured by p24 ELISA (Fig 5A) [24]. Most mutants showed similar levels of virus capture as WT, except for H12Q and H12Y mutants which were less efficiently captured (68% and 50%, respectively). Virus capture was undetected with the parental Raji cells lacking DC-SIGN.

When we assessed transmission of REJO WT and mutant viruses from DC-SIGN+ Raji cells to TZM.bl cells [24], all mutants showed decreased transfer relative to WT (Fig 5B). However, only the transfer of K2G, R15G, and all three H12 mutants (H12R, H12Q, H12Y) was reduced significantly. In the absence of DC-SIGN, no virus transmission was observed. The efficiencies of DC-SIGN-mediated capture and transmission correlated with Env incorporation to virions (Spearman r = 0.85, P = 0.002 and r = 0.64, P = 0.03, respectively) (Fig 5C), demonstrating the main contribution of Env expression level in determining virus interaction with DC-SIGN.

### Effects of SP mutations on REJO Env digestion by glycosidases

To determine if the SP mutations indeed alter sugar composition of the Env N-glycans, we sought more direct evidence of changes in N-glycan compositions on Env from 5 mutants displaying different patterns of Ab-mediated neutralization and DC-SIGN-mediated transmission. To this end, first we analyzed mobility shift of WT vs mutant virus-derived Env after digestion with Endo-H and PNGase F under reducing and non-reducing conditions. Endo H cleaves oligomannose residues at the β-1,4 linkage connecting two GlcNA residues and thus removes high mannose and hybrid but not complex N-glycans. PNGase F cleaves between asparagine and the first GlcNAc residue, and removes all N-glycans (high-mannose, hybrid and complex). The data show that under reducing condition Endo H digestion lowered the apparent molecular mass of WT and all five mutants to ~90 kDa bands that were reactive with gp120 MAbs (S5A Fig), while PNGase F digestion reduced the molecular mass to 90kD and 60 kDa (S5B Fig), similar to the pattern reported previously [25]. Endo H-digested K2G mutant migrated at a slightly lower rate, but no major differences were apparent between WT and SP mutants. Similar results were observed with Endo H digestion under non-reducing condition (S5C Fig). PNGase F digestion of WT and SP mutants under non-reducing condition also yielded Env products with comparable mobility, except that mutant H12Q exhibited more diffused wide bands as compared to WT and the other mutants (S5D Fig). Overall no dramatic changes were apparent in the proportions of high mannose and complex glycans on Env of SP mutants vs WT to significantly affect in their glycosidase digestion profiles.

### Effects of SP mutations on REJO Env reactivity with highly specific lectins

**Fig 6:**
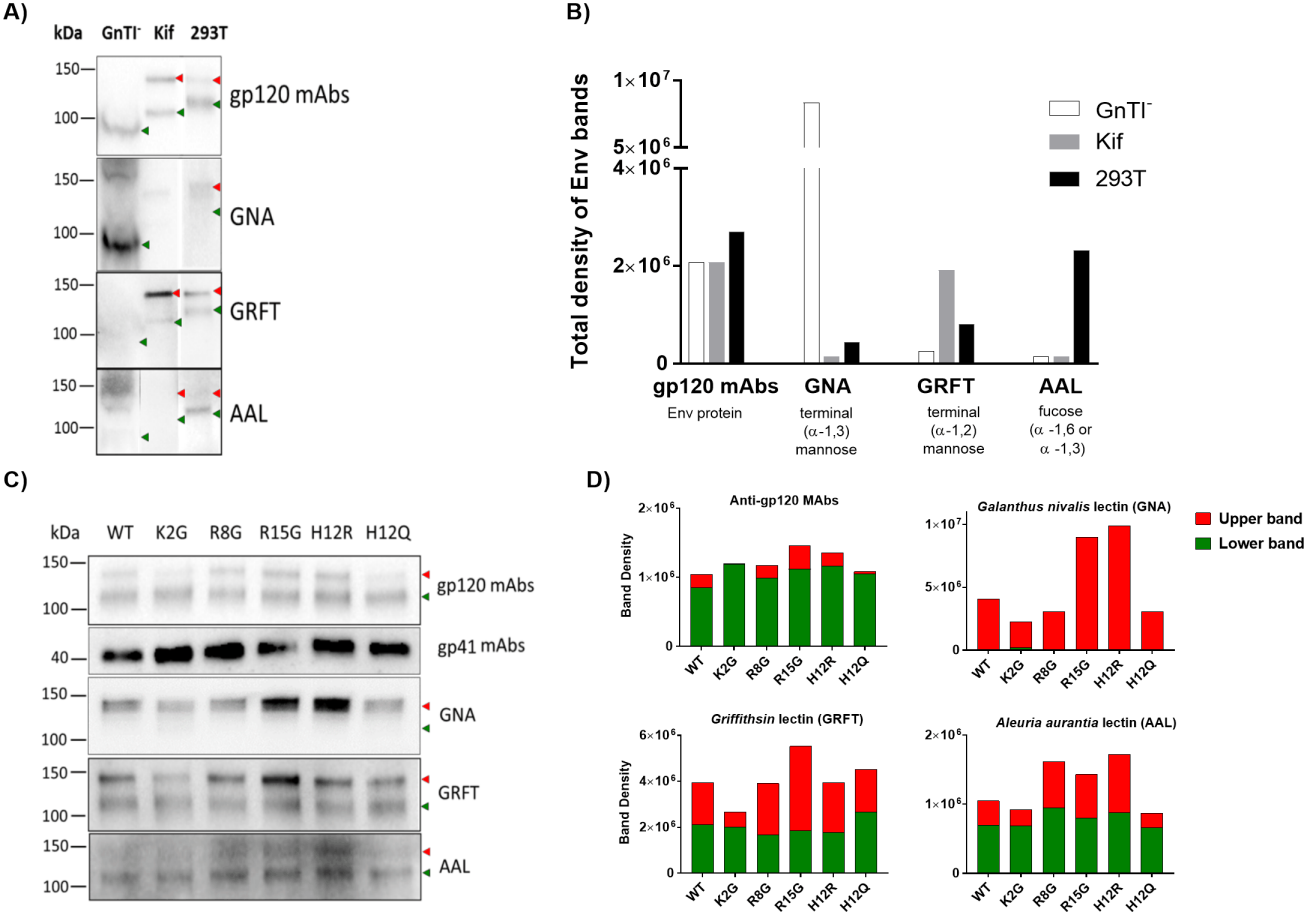
Analysis of REJO Env sugar moieties by lectin-probed Western blotting. The same amounts of Env from sucrose-pelleted virions were separated by SDS-PAGE (4–20%) under reducing conditions, blotted, and probed with an anti-gp120 MAb cocktail, an anti-gp41 MAb cocktail, and lectins (GNA, GRFT, and AAL). A) REJO WT virus produced in GnTI^-^ cells or in 293T cells in the presence vs absence of kifunensine (25μM) known to alter Env glycan compositions. B) Quantification of total density of Env bands from REJO WT shown in A). C) REJO WT and mutant viruses produced in 293T cells. D) Density measurements of the upper and lower Env bands as recognized by anti-gp120 MAbs and different lectins. Density analysis was done by Image Lab software. The two Env species with distinct molecular masses are indicated by red and green arrows.

To allow detection of finer changes in the sugar compositions of Env N-glycans from SP mutants vs WT, lectin-probed Western blot analyses were performed using lectins known to bind distinct sugar moieties: GNA (specific for terminal α1–3 mannose), GRFT (specific for α1–2 mannose) and AAL (specific to α-1,6 or a-1,3 fucose on complex glycans). The Env contents of sucrose-pelleted virus lysates were first quantified by Western blot using the anti-gp120 MAb cocktail similar to that done for Fig 1B, and equal amounts of WT and mutant Env were then used for the lectin-probed blots. The ability of lectins to detect differences in Env glycan compositions was first established by testing Env from REJO WT virus grown in 293T cells as compared to the same virus produced in HEK293S (GnTI^-^) cells and in 293T cells in the presence of 25μM kifunensine. Virus lysates were separated by SDS-PAGE and probed with anti-gp120 MAbs, GNA, GRFT and AAL. The gp120 MAb cocktail detected Env from all three viruses which displayed molecular mass differences consistent with the presence of different glycoforms (Fig 6A). The relative band intensities were quantified (Fig 6B). The GnTI^-^-derived virus expressed Env with mainly Man_5_GlcNAc_2_ N-glycans bearing terminal α1–3 mannoses; Env from this virus was well recognized by α1–3 mannose-specific GNA, but not by GRFT or AAL. Env of virus produced in presence of kifunensine, on the other hand, was enriched in Man_8-9_GlcNAc_2_ containing terminal α1–2 mannoses and was more reactive with GRFT than GNA or AAL. For comparison, the 293T-produced virus displayed Envs with various N-glycan types recognizable by GNA, GRFT and AAL. The data also revealed that, of the two Env bands present in the 293T-produced virus, the upper band corresponded to Env bearing high mannose-type glycans which reacted better with GNA and GRFT, whereas the lower band reacted more strongly with AAL indicating Env containing complex-type glycans.

**Fig 7:**
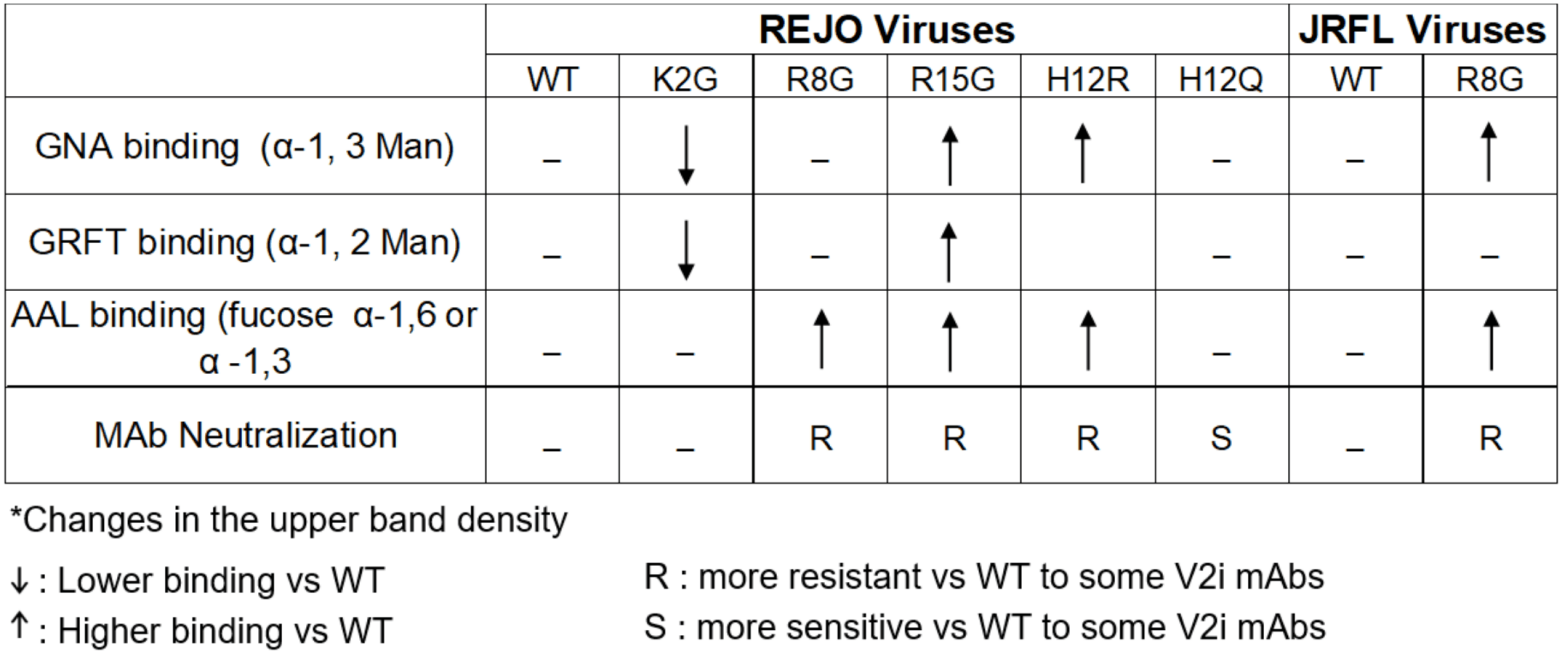
HIV phenotypes with Env SP mutation relative to WT*.

We subsequently utilized this assay for analyzing Env from REJO WT vs SP mutants produced in 293T cells. The data in Fig 6C and 6D showed that the upper and lower Env bands were detected in all 5 SP mutants and WT, and that comparable reactivity was seen with anti-gp120 MAbs, consistent with equivalent Env inputs. The amounts of gp41 were also similar for all viruses. However, lectin binding showed distinct patterns among the SP mutants. The terminal α1–3 mannose-specific GNA, which detected only the upper band, reacted more strongly to R15G and H12R as compared to WT and the other SP mutants. GRFT, specific for terminal α1–2 mannose, reacted to both upper and lower bands, but showed stronger binding to the upper band of R15G and weaker binding to that of K2G as compared to WT. The fucosylated glycan-binding AAL detected mainly the lower bands for WT, K2G, and H12Q, but had enhanced binding to the upper bands of R8G, R15G and H12R. As summarized in Fig 7, differences in lectin binding to Env of SP mutants were evident to indicate enrichment of certain oligomannose- and fucosylated complex-types of N-glycans on Env of SP mutants. For examples, R15G and H12R had higher levels of terminal α1–3 mannoses and fucosylated glycans than WT and the other mutants. R15G also had higher levels of terminal α1–2 mannoses. In contrast, R2G had lower levels of terminal α1–3 and α1–2 mannoses.

**Fig 8:**
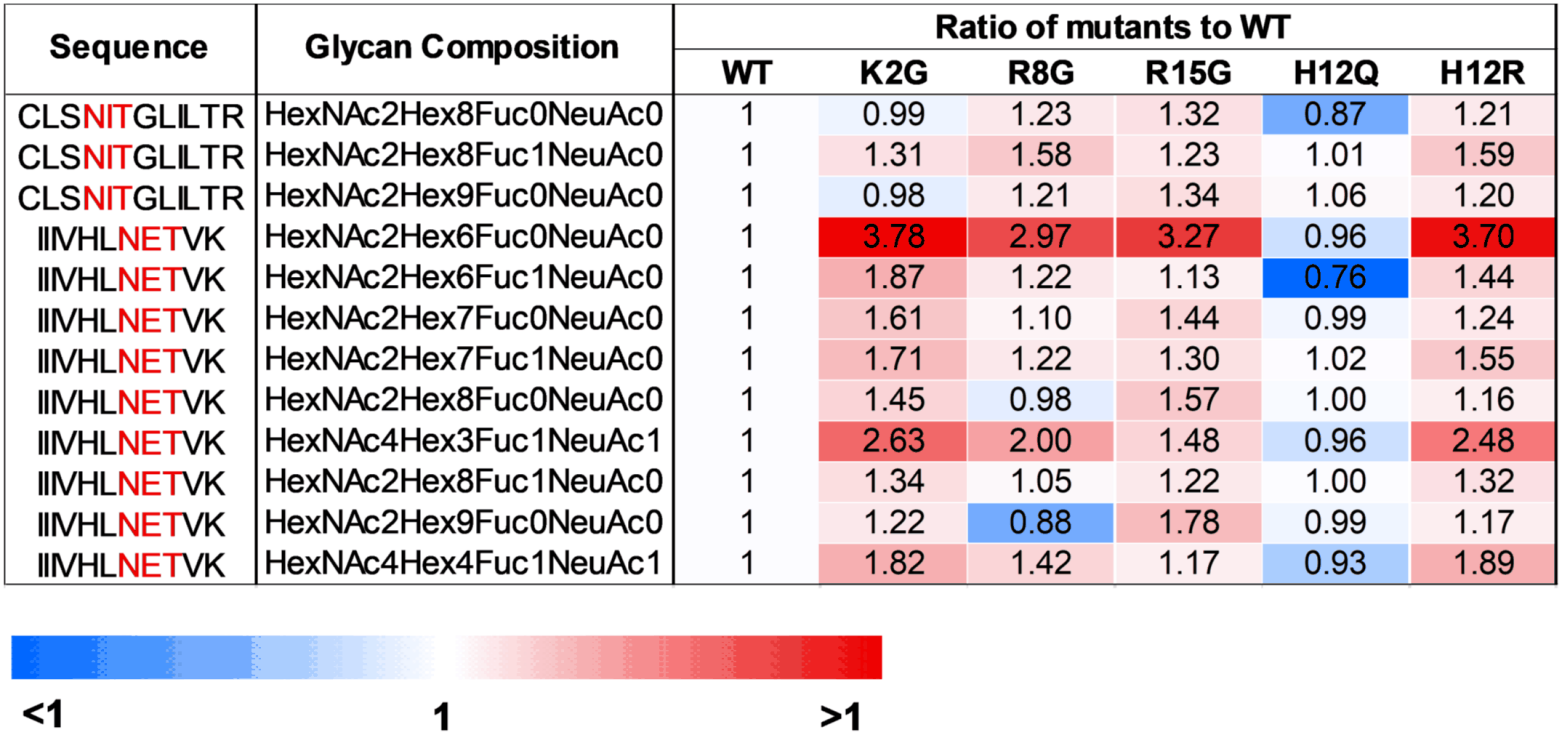
Proteomic and glycoproteomic analyses of virion proteins using liquid chromatography-tandem mass spectrometry (LC-MS/MS). Sucrose-pelleted virions were denatured with 8M in1 M ammonia bicarbonate buffer. The denatured proteins were than prepared for trypsin digestion at 37°C overnight. The samples containing peptides were acidified pH = 3 and desalted using C18 SPE column. The C18 elute was dried in the speed-vac and then resuspended in 0.2% formic acid. The samples (1 μg) were then subjected to LC-MS/MS. Relative abundance of different glycoforms found on 2 identified glycopeptides from SP mutants vs WT were calculated and shown as ratio of mutant to WT.

The glycan composition of sucrose-pelleted REJO WT and mutant viruses was also evaluated by liquid chromatography–mass spectrometry (LC-MS/MS). Using SEQUEST with 1% FDR for assignment of spectra to peptides, we were able to identify peptides derived from REJO GAGpr55, Pol and Env with sequence coverage of 75%, 58% and 29%, respectively (S2 Table). We also detected 2 intact glycopeptides, IIIVHLN^290^ETVK and CLSN^446^ITGLILTR, corresponding to REJO Env positions 283–293 (C2) and 445–456 (C4). These Env fragments displayed 9 and 3 different glycoforms, respectively (Fig 8 and S3 Table). Importantly, the analysis clearly showed alterations in the relative abundance of glycoforms associated with SP mutants vs WT. Thus, the LC-MS/MS data supports the idea that a single change in the SP can indeed alter the sugar composition of the Env N-glycans, which in turn influenced virus interaction with DC-SIGN and virus neutralization by Abs.

### Effect of SP mutations is not virus isolate specific

**Fig 9:**
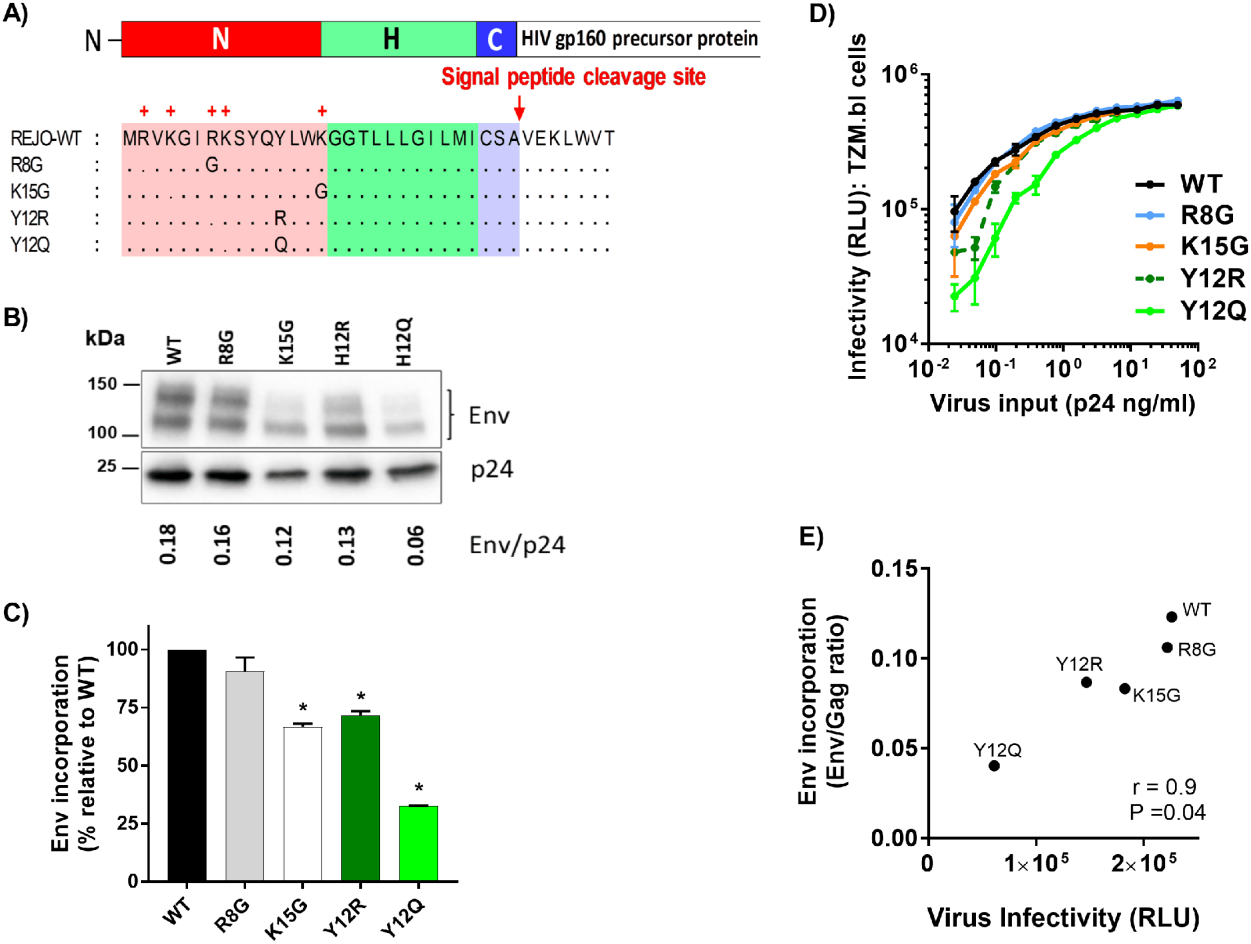
Effects of SP mutations on JRFL Env expression, virus infectivity and reactivity to different MAbs. (A) Schematic representation of JRFL WT and four different SP mutations evaluated in this study. (B) Measurement of Env incorporation by Western blot. JRFL WT and mutant viruses were produced in transfected 293T cells, lysed, and analyzed by SDS-PAGE (4–20%) and Western blot. An anti-gp120 MAb cocktail (V3: 391/95-D, 694/98-D, 2219, 2558; C2: 847-D, 1006-30D; C5: 450-D, 670-D) and a p24 Gag MAb (91–5) were used to detect the relative levels of Env and Gag associated with virions. The ratios of Env/Gag were calculated. (C) The levels of Env incorporation into JRFL mutant virions relative to that of WT were calculated based on their Env/Gag ratios (WT value was set to 100%). *, p< 0.01 (ANOVA). (D) Infectivity of JRFL WT vs. mutant viruses in CD4+ TZM.bl cells exposed to titrated viruses with equivalent p24 contents. (E) Correlation of virus infectivity in CD4+ TZM.bl cells with Env incorporation into the virions by Spearman’s rank test. Virus infectivity was based on RLU produced upon infection with a fix amount of virus input (0.9 ng p24/ml).

To assess whether the SP mutations can impart similar effects on another HIV-1 isolate, we mutated charged residues in the SP of JRFL Env, a subtype B chronic isolate. We substituted the R/K residues at positions 8 and 15 to glycine (R8G, K15G) (Fig 9A). In addition, we introduced Y12Q and Y12R mutations, because JRFL has a Y residue at position 12 instead of H. Similar to the effect seen on REJO, SP mutations K15G, Y12R and Y12Q reduced JRFL Env incorporation into virions, whereas R8G mutation had no effect (Fig 9B and 9C). The Y12R and Y12Q mutants also showed reduced infectivity detectable at lower p24 inputs, while the infectivity of R8G and K15G was comparable to WT (Fig 9D). Correlation was observed between Env incorporation and virus infectivity (*r* = 0.9, *P* = 0.04 by Spearman test) (Fig 9E). Effect of the SP mutations on antigenicity was assessed by testing solubilized gp120 proteins from JRFL WT and mutant viruses in ELISA. As shown in S6A and S6B Fig, the SP mutations did not abolish gp120 reactivity to MAbs tested. However, gp120 from the K15G, Y12R and Y12Q mutants reacted more weakly with most V2i MAbs, and had reduced binding to CD4-IgG2. The reactivity with V3 MAbs was minimally affected. Interestingly, the JRFL R8G mutant showed increased binding to many MAbs tested, including V2i MAbs (697, 1357), V3 MAbs (3074, 3869), and to CD4-IgG2 (S6B Fig); such increase in ELISA reactivity was not seen with REJO R8G and other REJO mutants (S4B Fig).

**Fig 10:**
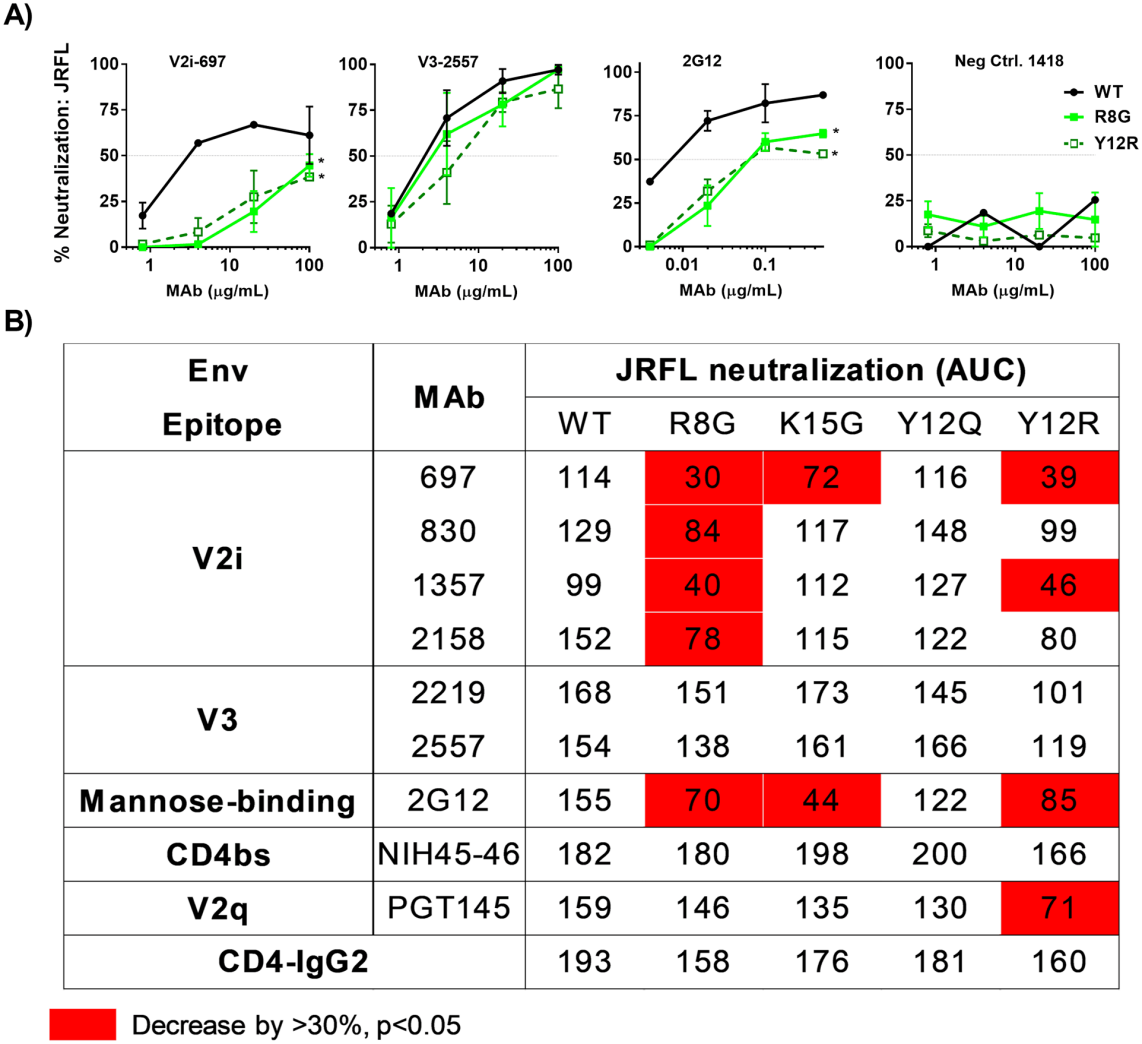
Neutralization of JRFL WT and mutant viruses by different MAbs and CD4-IgG2. Neutralization assays were performed for each of MAb-virus pairs as described in Fig 2. AUC values were calculated from titration curves. A) Titration curves of representative virus-MAb pairs. B) JRFL WT and mutant neutralization by MAbs targeting V2i, V3, V2q, and the CD4bs and by CD-IgG2. AUC values that decreased by >30% and had p<0.05 relative to WT are shown in red. Means and standard errors from two to three experiments are shown.

Next, we evaluated the neutralization phenotype of the JRFL Env SP mutants. Except for Y12Q mutation that did not affect JRFL neutralization, the SP mutations increased JRFL resistance to V2i MAbs (Fig 10). R8G mutant was the most resistant to all 4 V2i MAbs tested (Fig 10), although the V2i MAb reactivity with R8G gp120 was comparable or even increased (S6 Fig). In contrast, the SP mutations minimally affected JRFL neutralization by V3 and CD4bs MAbs. Overall, 7/16 (44%) mutant-V2i MAb combinations showed increased resistance, while no mutant (0/8) became more resistant to V3 MAbs. This pattern was similar to that seen with REJO (Figs 3 and 4 and S7 Fig for side-by-side comparison of REJO and JRFL mutants). Moreover, the same 3 SP mutations affecting V2i MAb neutralization (R8G, K15G and Y12R) rendered JRFL more resistant to the mannose-binding MAb 2G12. These data indicate that these SP mutations induce alterations in the N-glycan oligosaccharide composition that influence virus sensitivity to neutralizing Abs [26, 27].

**Fig 11:**
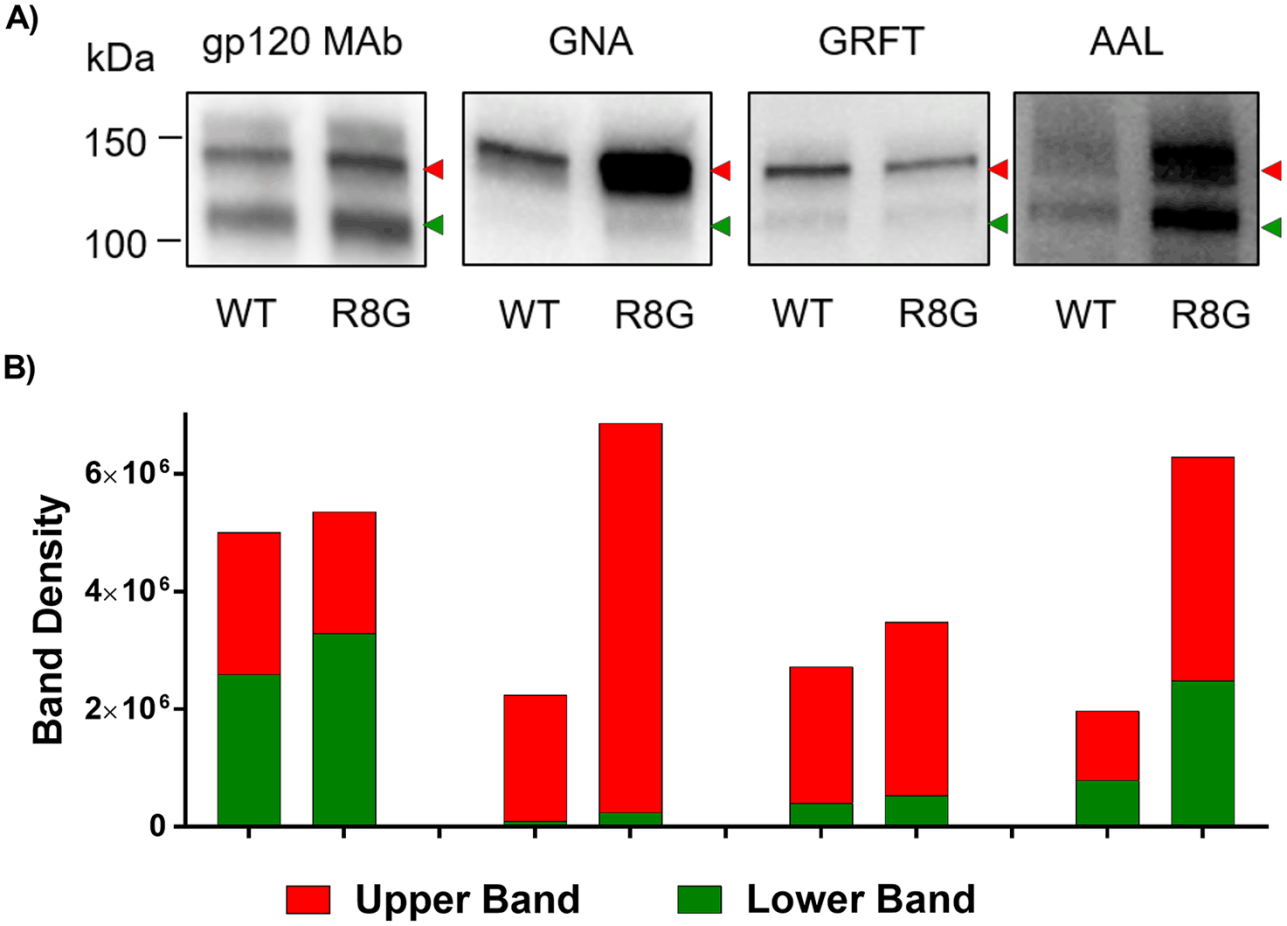
Analysis of JRFL Env sugar moieties by lectin-probed Western blotting. The same amounts of Env from JRFL WT and mutant viruses produced in 293T cells were separated by SDS-PAGE (10%) under reducing condition, blotted, and probed with an anti-gp120 MAb cocktail or lectins (GNA, GRFT, and AAL). A) Env reactivity with MAbs versus lectins. Two Env species with distinct molecular masses are indicated by red and green arrows. B) Density measurements of the upper and lower Env bands as recognized by anti-gp120 MAbs and different lectins. Density analysis was done by Image Lab software.

The glycan composition of JRFL R8G mutant, which showed the most altered neutralization pattern, was further compared to its WT counterpart in lectin-probed Western blots. Terminal α1–3 mannose-specific GNA and terminal α1–2 mannose-specific GRFT detected only the upper band of JRFL Env, while fucose-binding AAL was reactive with both upper and lower bands (Fig 11). GNA and AAL reacted more strongly with the JRFL R8G mutant as compared to WT, whereas GRFT binding was comparable, demonstrating specific enrichment of N-glycans with terminal α1–3 mannose and fucose moieties on JRFL R8G Env (Figs 11 and 7). These results provide corroborating evidence that single amino-acid substitutions in the Env SP are sufficient to influence the oligosaccharide composition of the Env N-glycans on different HIV-1 isolates to result in altered virus phenotypes.

## Discussion

This paper evaluated the influence of HIV-1 Env SP in modulating the phenotypic characteristics of HIV-1 viruses in the context of IMCs (REJO and JRFL), in which natural linkages to all regulatory and structural proteins were maintained [28, 29]. The REJO and JRFL Env SPs carry 6 and 5 positively charged residues, respectively. We sought to understand the importance of these charged residues in determining virus phenotypes and Env functions. The results show that mutating one of these charged residues was sufficient to alter Env incorporation to virions, virus binding and transmission via DC-SIGN, virus neutralization by MAbs, and oligosaccharide compositions of Env glycans. These single substitutions did not completely abrogate virus infectivity, but mutations affecting position 12 (REJO: H12R, H12Q, H12Y; JRFL: Y12R, Y12Q) and position 15 (REJO: R15G; JRFL: K15G) significantly decreased Env packaging into the virions and also affected virus infectivity. Notably, enrichment of H at position 12 was identified as a signature of acute HIV-1 isolates [14, 15]. A similar signature site was also reported on the acute SIV Env SP [16]. The presence of H or R at this position was associated with higher Env expression and virion incorporation levels [15]. In contrast to this literature, our results with mutant H12R showed lower Env incorporation and slight decrease in infectivity, relative to WT, which may be due to the differences in the choice of virus strains and the use of IMCs as opposed to pseudoviruses. However, consistent with this past study, lower Env incorporation was observed in virions when non-signature amino acids Q or Y were introduced at this position as compared to amino acids H or R. The molecular basis for these changes remains unclear. During protein targeting, the basic residues in the cationic N-region of the SP are suggested to establish an electrostatic interaction with the phosphate backbone of the signal recognition particle (SRP) [6, 30] that influences the subsequent binding to the Sec61p complex [31]. Hence, a possible explanation for the alterations of Env expression by the SP mutations is that the basic residue removal affects the SP binding to SRP and consequently the rate of Env transport to or processing in the ER and the Env glycosylation. Nonetheless, we found no evidence for delayed rate of gp120 synthesis as a result of SP mutations. No Env accumulation was detected in the cells, either. Moreover, as indicated by the H12R mutation, removal of basic residue alone cannot fully explain the SP mutant phenotypes. Rather, H12Q and H12Y mutations drastically diminished the overall Env expression on the virions and also in the cells. Altogether, these data support the notion that the signature amino acids at position 12 of the Env SPs determine virus infectivity and transmissibility by controlling Env expression and incorporation to virions.

Among the REJO and JRFL SP mutations studied, mutations that increased virus resistance to V2i MAbs were located at residues 8, 12, and 15. The upstream REJO K2G mutation led to increased Env being incorporated into the virion, but did not alter REJO neutralization sensitivity to any of the MAbs tested. However, considering that mutations at some of these residues affected DC-SIGN-mediated uptake and transmission, and that viruses with altered N-glycan compositions as a result of glycosidase inhibitor or lack of glycosyltransferase enzyme also displayed increased resistance to V2i MAbs, these SP mutations most likely altered the oligosaccharide profiles of the Env N-glycans; this was also consistent with the lectin-probed Western blot and LC-MS/MS data. The V2i epitopes themselves do not contain N-glycans [32], but the MAb recognition of V2i epitopes are dependent on N-glycans [26]. The reactivity of the V2i MAb 697, for example, was abrogated by treating Env with sodium metaperiodate that oxidizes oligosaccharides [26]. The V2i MAb 2158 was sensitive to mutations that removed the N190-glycan, reducing the MAb binding to 20–50% [27]. Single mutations at other PNGs on gp120 also induced global structural changes that better exposed V2i, V3, and the CD4-binding site to yield more sensitive viruses [33]. To our best knowledge, our study is the first to reveal that a single amino acid change increases HIV-1 resistance to neutralization by MAbs, particularly V2i MAbs. Remarkably, the changes uniquely involve the Env SP, an Env fragment not present in the mature Env displayed on the virions. Our LC-MS/MS analysis did not detect any SP fragments associated with REJO WT or SP mutant virions. Nonetheless, unlike the complete loss of V2i MAb-mediated neutralization instigated by GnTI- cells or kifunensine that inflicts wide-spread and more homogenous effects on all 29 N-glycans potentially present on the REJO Env, the single-point SP mutations studied here cause more subtle changes in the oligosaccharide moieties detectable by differential reactivity with specific lectins and by mass spectrometry. Increased resistance to neutralization by V2i MAbs was also seen when mutations were made in the SP of JRFL Env, indicating that the effects are not isolate-specific. Moreover, increased resistance to MAb 2G12, which specifically recognizes high-mannose glycans on HIV-1 Env, was observed with JRFL mutants R8G, K15G and Y12R, further strengthening the evidence that these SP mutations affects oligomannose moieties of the Env glycans.

The SP has been implicated in governing HIV-1 Env glycosylation [6]. Our data from lectin-probed Western blotting and LC-MS/MS analyses provided direct evidence for altered oligosaccharide contents of Env glycans as a result of SP mutations. Increased binding to fucose-specific AAL was demonstrated by R8G, H12R and R15G, all of which rendered the REJO virus resistant to V2i MAbs (Fig 7). R15G and H12R, but not R8G, also reacted more strongly with GNA, a lectin specific for terminal α1–3 mannoses present on Man_5-8_ and hybrid glycans but not on Man_9_ and complex glycans [34, 35]. Similarly, the JRFL R8G mutant also showed increased binding to GNA and AAL as compared to its WT counterpart. In contrast, REJO K2G, which display comparable neutralization as WT, had no change in its fucose content as detected by AAL, and had lower reactivity with GNA and GRFT. Taken together, the data demonstrate that increased α1–3 or α1–2 mannoses and fucose contents of HIV-1 Env are associated with increased virus resistance to neutralization by V2i Abs. The REJO H12Q mutant, on the other hand, showed increased neutralization sensitivity to some of the V2 and V3-specific MAbs. The reason for this phenotype is unclear, but this mutant showed no discernable changes in lectin binding, although apparent alterations were noted with its gp120-MAb reactivity and glycosidase digestion under non-reducing condition. The LC-MS/MS results also show similar glycoform profiles for H12Q vs WT, although changes on other glycosylation sites unidentified in this study cannot be ruled out.

In conclusion, this study shows that mutations in the Env SP impact Env incorporation into HIV-1 virions, Env binding to lectins, virus transmission via DC-SIGN, and virus susceptibility to neutralization by MAbs. The study also provides evidence that the Env SP serves as a modulator of Env glycosylation to influence Env function and immune recognition.

## Materials and methods

### Cell lines and plasmids

HEK293T/17 and HEK293S (GnTI^-^) cells were obtained from the American Type Culture Collection (ATCC). The following reagents were obtained through the NIH AIDS Reagent Program, Division of AIDS, NIAID, NIH: TZM-bl from Dr. John C. Kappes, Dr. Xiaoyun Wu and Tranzyme Inc [36]; Raji and Raji/DC-SIGN cells from Drs. Li Wu and Vineet N. KewalRamani [37] and pREJO.c/2864 (cat# 11746) from Dr. John Kappes and Dr. Christina Ochsenbauer.[38]. pNL-JRFL (NFN-XS-r-HSA) was constructed by Dr. Jerome Zack (UCLA) [39]

### Human monoclonal antibodies

V2i, V3, and control MAbs used in this study were produced in our laboratory as described [26, 40–45]. The following antibody reagents were obtained through the NIH AIDS Reagent Program, Division of AIDS, NIAID, NIH: anti-HIV-1 gp120 Monoclonal (IgG1 b12) from Dr. Dennis Burton and Carlos Barbas [46]; anti-HIV-1 gp120 Monoclonal (2G12) from Dr. Hermann Katinger [47]; NIH 45–46 from Pamela Bjorkman [18], anti-HIV-1 gp120 Monoclonal PG16 [48] and anti-HIV-1 gp120 Monoclonal PGT145 [49]. V2q MAb PG9 was purchased from Polymun Scientific. V2i MAbs target V1V2 epitopes that overlap with the integrin α4β7-binding motif, while the V2q MAb PG9 is specific for a quaternary V1V2 epitope preferentially presented on the Env trimer [17, 27].

### Plasmid constructs and virus production

Single-point mutations were introduced to the Env SPs of pREJO.c/2864 and pNL-JRFL infectious molecular clones (Figs 1A and 9A) by multi-step overlapping PCR mutagenesis strategy using Pfx50^**™**^ DNA Polymerase PCR System (Invitrogen). In the first PCR step, mutated fragments were individually generated in two separate reactions using two primer pairs. The list of all primer pairs is shown in S1 Table. For example, the primers AvrIIF/K2GR and K2GF/BstEIIR were used to generate the mutant K2G. The fragments were agarose gel-purified, combined, and added to a second-stage PCR with the flanking primers AvrIIF and BstEIIR. Products of the second-stage PCR were digested by AvrII and BstEII restriction enzymes and inserted into the AvrII- and BstEII-digested fragment of pREJO.c/2864 to yield the mutant K2G. The other mutants were similarly constructed using their respective primers. In case of JRFL the second-stage PCR product was digested with EcoRI and NheI and inserted into the EcoRI- and NheI-digested fragment of pNL-JRFL. All the plasmids were sequenced to confirm the presence of the desired sequence changes without any other mutations.

Viruses were produced by transfecting 293T/17 cells with wild type (WT) or mutated pREJO.c/2864 and pNL-JRFL plasmids using jetPEI transfection reagent (Polyplus). Glycan-modified viruses were generated in the presence of 25μM kifunensine or by transfecting GnTI^-^ cells. Supernatants were harvested after 48 hrs and clarified by centrifugation and 0.45μm filtration. Single-use aliquots were stored at −80°C. Viruses were sucrose pelleted as in [15]. Virus infectivity was assessed on TZM.bl cells as described [50]. An HIV-1 p24 enzyme-linked immunosorbent assay kit (XpressBio) was used to quantify the p24 content in supernatants using the manufacturer’s protocols.

### Western blot analyses with antibody and lectin probes

To quantify the ratios of Env to p24 proteins incorporated into the WT and mutant viruses and to evaluate Env reactivity with different lectins, Western blot analyses were performed. The virus particles pelleted from 200μl supernatant were lysed, resolved by SDS-PAGE on 4–20% tris-glycine gels (Bio-Rad), and blotted onto membranes, which were then probed with antibodies or lectins. A cocktail of anti-human anti-gp120 MAbs (anti-V3: 391, 694, 2219, 2558; anti-C2: 841, 1006; anti-C5: 450, 670, 722; 1μg/ml each), a cocktail of anti-gp41 MAbs (181-D, 240-D, 246-D, 167–7, 1367, 2295, 2556; 1μg/ml each), and anti-gp41 MPER MAb 2F5 (2μg/ml) were used to detect Env. MAb 91-5D (1μg/ml) was used to detect Gag p24. His tagged-GRFT (Griffithsin lectin; NIH AIDS repository), biotinylated GNA (*Galanthus nivalis* lectin; Vector Laboratories), and biotinylated AAL (*Aleuria aurantia* lectin; Vector Laboratories) were each used at 2μg/ml. Lectin binding was detected with HRP-neutravidin (1:1500 for 1 hr RT). For GRFT, the blots were incubated with anti-mouse His-tag MAb (1:1000 for 1hr) followed by anti-mouse HRP (1:1000 for 1hr RT). All dilutions were made in Superblock T20 (SuperBlock T20 (PBS) Blocking Buffer; Thermofisher). Membranes were developed with SuperSignal West Pico reagents (Pierce) and scanned by a ChemiDoc Imaging Systems (Bio-Rad Laboratories). Purified recombinant gp120 and p24 proteins were also loaded at a known concentration as controls and quantification standards. Band intensities were quantified using the Image Lab Software Version 5.0 (Bio-Rad).

### gp120–MAb binding assay

The relative binding of MAbs to gp120 from the WT and mutant viruses was measured by a sandwich ELISA. ELISA plates were coated with the sheep anti-gp120 Abs (D7324; 2μg/ml, Aalto BioReagents, Dublin, Ireland), blocked with 2% bovine serum albumin (BSA) in phosphate-buffered saline (PBS), and incubated with Env (20 ng/ml as measured by Western blots) from 1% Triton X-treated virus lysates. Serially diluted MAbs (0.01–10μg/ml) were then added for 2hrs, and the bound MAbs were detected with alkaline phosphatase-conjugated goat anti-human IgG and p-nitrophenyl phosphate substrate.

The kinetics of Env production in the cells was measured similarly by ELISA. Briefly, HEK293T cells in 6 well-plates were transfected with REJO WT or mutant plasmids. Samples containing cells and supernatants were frozen at -80°C at 2, 8, 12, 24, 28, and 36 hrs post-transfection. Samples were clarified by centrifugation, lysed with 1% Triton-X, and tested in the sandwich ELISA. Env were captured by polyclonal sheep anti-C5 antibodies and probed with MAb EH21 (specific for C1) or MAb A32 (specific for a discontinuous epitope involving residues within the C1, C2 and C4 regions).

### Neutralization assay

Virus neutralization was measured with TZM.bl target cells using a β-galactosidase-based assay (Promega) [17, 51]. Because REJO and JRFL neutralization by anti-V3 and anti-V2i MAbs are attained only after >18 hr pre-incubation of the virus-MAb mixture [17], in this study neutralization assays were performed with 24 hrs of pre-incubation for all MAbs, except for PG9, PGT145 and CD4-IgG2 which were tested with the standard 1 hr incubation. Each condition was tested in duplicate or triplicate. Percent neutralization was determined based on virus control (TZM.bl cells with virus alone) and cell control (TZM.bl cells only) under the specific assay condition. Virus inputs corresponding to 150,000–200,000 RLUs were used.

### DC-SIGN-mediated virus capture and transmission assays

For virus capture assay, parental Raji or DC-SIGN+ Raji cells (1x10^6^ each) were incubated for 2 hrs with WT or mutant REJO viruses (15 ng/ml p24). After unbound viruses were removed by washing, the cells were lysed in 1% Empigen detergent for 1 hr at 56°C, and p24 levels were determined by ELISA (XpressBio). For transmission experiments, Raji or DC-SIGN+ Raji cells (1x10^5^) were incubated for 2 hrs with virus (3 ng/ml p24), washed three times and co-cultured with TZM-bl cells for 48 hrs in the presence of DEAE. HIV-1 transmission to TZM.bl cells was quantified by measuring β-galactosidase activity (Promega).

### Proteomic and glycoproteomic analyses of virion proteins using liquid chromatography-tandem mass spectrometry (LC-MS/MS)

The LC-MS/MS was performed as previously [52]. Briefly, sucrose-pelleted virions were added to 8 M urea in 1 M ammonium bicarbonate buffer and reduced with 5 mM DTT at 37°C for 1 h. Proteins were alkylated by iodoacetamide at a final concentration of 10 mM and incubated at RT in the dark for 40 min. Samples were applied to the Microcon-10 kDa centrifugal filter unit and centrifuged until the solution was minimal in the filter unit. The samples were washed six times with 0.1 M ammonium bicarbonate buffer, and 5 μg of trypsin was added in the buffer after the final wash. The digestion was incubated at 37°C for overnight. The tryptic peptides were harvested by centrifugation. The solution containing peptides and glycopeptides were acidified to pH = 3, desalted by C18 cartridge according to manufacturer’s instructions, dried in a speed-vac, and resuspended in 0.2% formic acid. The samples (1 μg) were separated through a Dionex Ultimate 3000 RSLC nano system (Thermo Scientific). MS analysis was then performed using a Thermo Q Exactive mass spectrometer (Thermo Scientific). HIV-1 peptides were identified by using SEQUEST in Proteome Discoverer software (Thermo Fisher Scientific, version 2.2). The intact Env glycopeptides were identified using GPQuest [52, 53]. To quantify the glycosylation in different mutants label-free quantification of Env peptides and glycopeptides was done using Thermo SIEVE software version 2.1 (S3 Table). The data was normalized using a non-glycosylated the Env peptide VVQIEPLGIAPTR that showed high confident identification and most reliable quantitative value in the LC-MS/MS data. Normalization was validated using two other Env peptides (LTPLCVTLK and EATTTLFCASDAK).

### Statistical analysis

Comparisons of virus neutralization and MAb binding were performed using GraphPad Prism. Statistical analyses were performed on neutralization data that reached ≥50%.

## Supporting information

S1 Table Oligonucleotides used for construction of mutant plasmids. (XLSX)

S2 Table Identification of peptides from HIV GAGPr55, Pol and Env gp120. (XLSX)

S3 Table Identification and quantitation of intact glycopeptides from REJO WT and SP mutants. (XLSX)

S1 Fig Sequence variability of Env SPs from reference HIV-1 isolates of different subtypes and CRFs. Source: Los Alamos HIV Database.
(TIF)

S2 Fig Effects of SP mutations on REJO Env expression in the cells. (A) Env expression in the cells as detected by an anti-gp120 MAb cocktail. WT and mutant REJO constructs were transfected into 293T cells. The cells were washed with PBS, lysed, and analyzed by Western blot. An anti-gp120 MAb cocktail (V3: 391/95-D, 694/98-D, 2219, 2558; C2: 847-D, 1006-30D; C5: 450-D, 670-D) was used as a probe. GADPH was used as loading control. (B) Expression of mutant Env in the cells relative to WT (100%).*, p< 0.01 (ANOVA). (C) Rate of Env production in cells as measured by MAb EH21 specific for a linear C1 epitope and MAb A32 specific for a conformation-dependent epitope involving C1, C2 and C4. 293T cells were transfected by REJO WT or mutant plasmids and harvested from 2 to 36 hrs after transfection. Env was captured onto ELISA plate by anti-C5 polyclonal sheep antibody and reacted with MAbs EH21 or A32.
(TIF)

S3 Fig gp41 expression in sucrose-pelleted virions. Western blots prepared as in Fig 6C were probed with A) anti-gp41 MAb cocktail (181-D, 240-D, 246-D, 167–7, 1367, 2295, 2556; 1μg/ml each), or B) gp41 MPER-specific MAb 2F5 (2 μg/ml).
(TIF)

S4 Fig Reactivity of REJO gp120 from WT and SP mutants with MAbs to V2i, V3, and the CD4bs and with CD4-IgG2. Virus lysates in 1% Triton-X100 containing equivalent constant amounts of gp120 from WT and mutant viruses were added to ELISA wells coated with sheep anti-C terminal gp120 antibodies and the captured gp120 proteins were reacted with MAbs or CD4-IgG2. The MAbs were titrated ten-fold from 10 μg/ml, while CD4-IgG2 was titrated five-fold from 10 μg/ml. A) Titration curves from representative MAb-virus pairs were shown. B) AUC values were calculated from all titration curves and the decreased levels of MAb binding to mutant gp120 versus WT were color-coded.
(TIF)

S5 Fig Mobility shift of virus-derived Env after glycosidase digestion. Sucrose-pelleted REJO WT and mutant virions were treated by Endo H or PNGase F under reducing (A and B respectively) or native non-reducing conditions (C and D respectively). All samples were then run on SDS-PAGE (10%) under reducing condition, and the blots were probed with anti-gp120 MAb cocktail. Untreated REJO WT (UT) was included for comparison. Red dotted lines are shown to highlight the observed changes in mobility shift of WT vs mutant Env proteins.
(TIF)

S6 Fig Reactivity of gp120 from JRFL WT and SP mutants with MAbs to V2i, V3, and the CD4bs and with CD4-IgG2. (A) Titration curves from representative MAb-virus pairs showing reactivity of gp120 from JRFL WT and SP mutant viruses with MAbs to V2i, V3, and the CD4bs and with CD4-IgG2. Virus lysates in 1% Triton-X100 containing equivalent constant amounts of gp120 from WT and mutant viruses were added to ELISA wells coated with sheep anti-C terminal gp120 antibodies and the captured gp120 proteins were reacted with MAbs or CD4-IgG2. The MAbs were titrated ten-fold from 10 μg/ml. B) AUC values were calculated from all titration curves and color-coded to show increased or decreased levels of MAb binding to mutant gp120 versus WT.
(TIF)

S7 Fig Comparison of neutralization of REJO and JRFL SP mutants by different MAbs and CD-IgG2. AUC values from Figs 3 and 10 are presented together to show the comparable effects of analogous SP mutations on neutralization of REJO and JRFL.
(TIF)
