# Supplementary figures and images for: Alterations of HIV-1 envelope phenotype and antibody-mediated neutralization by signal peptide mutations
Source: PLoS Pathog. 2018 Jan 25;14(1):e1006812. doi: 10.1371/journal.ppat.1006812 (PMC5800646; doi:10.1371/journal.ppat.1006812)

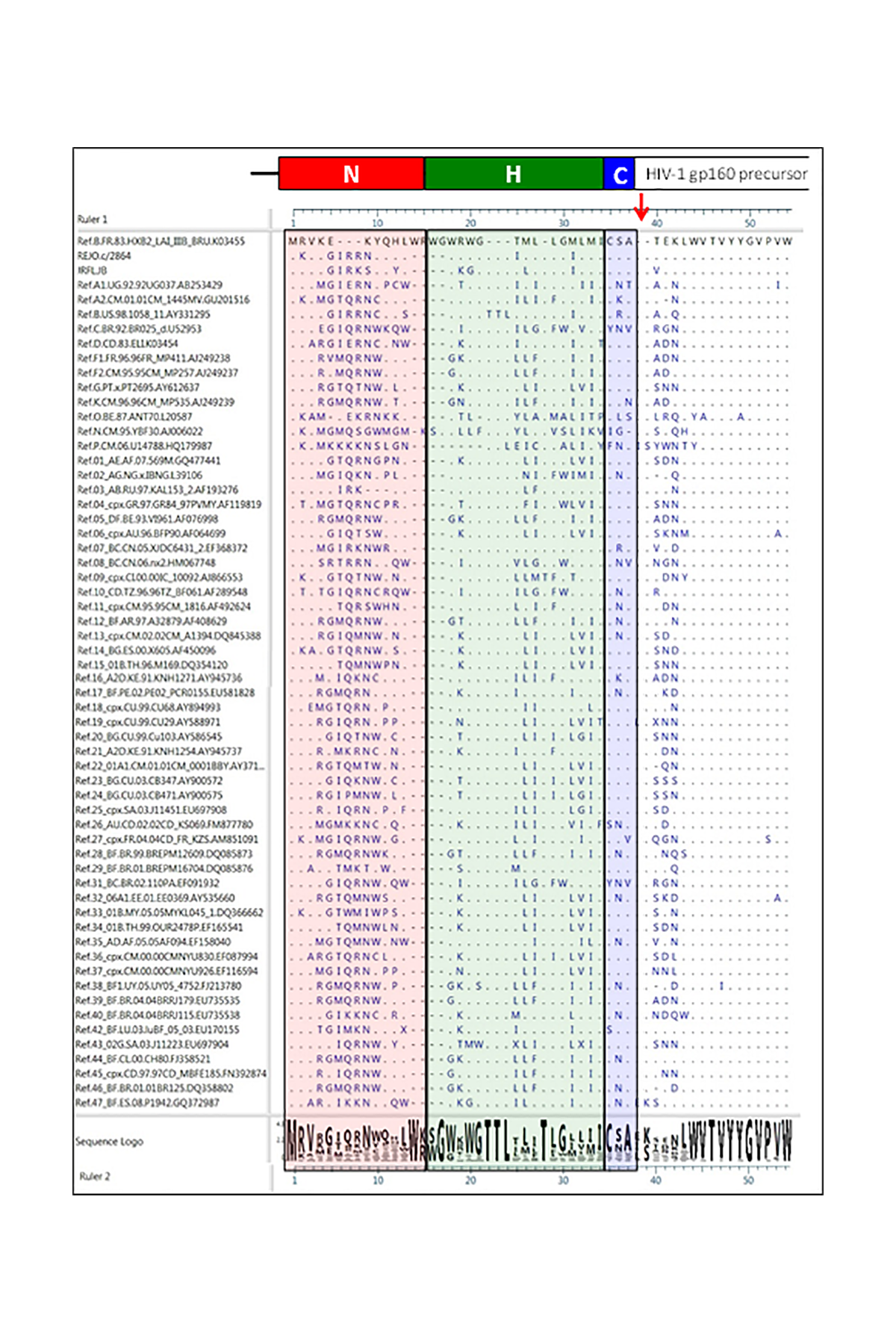

Supplement: S1 Fig — Source: Los Alamos HIV Database. (TIF) [file ppat.1006812.s004.tif]

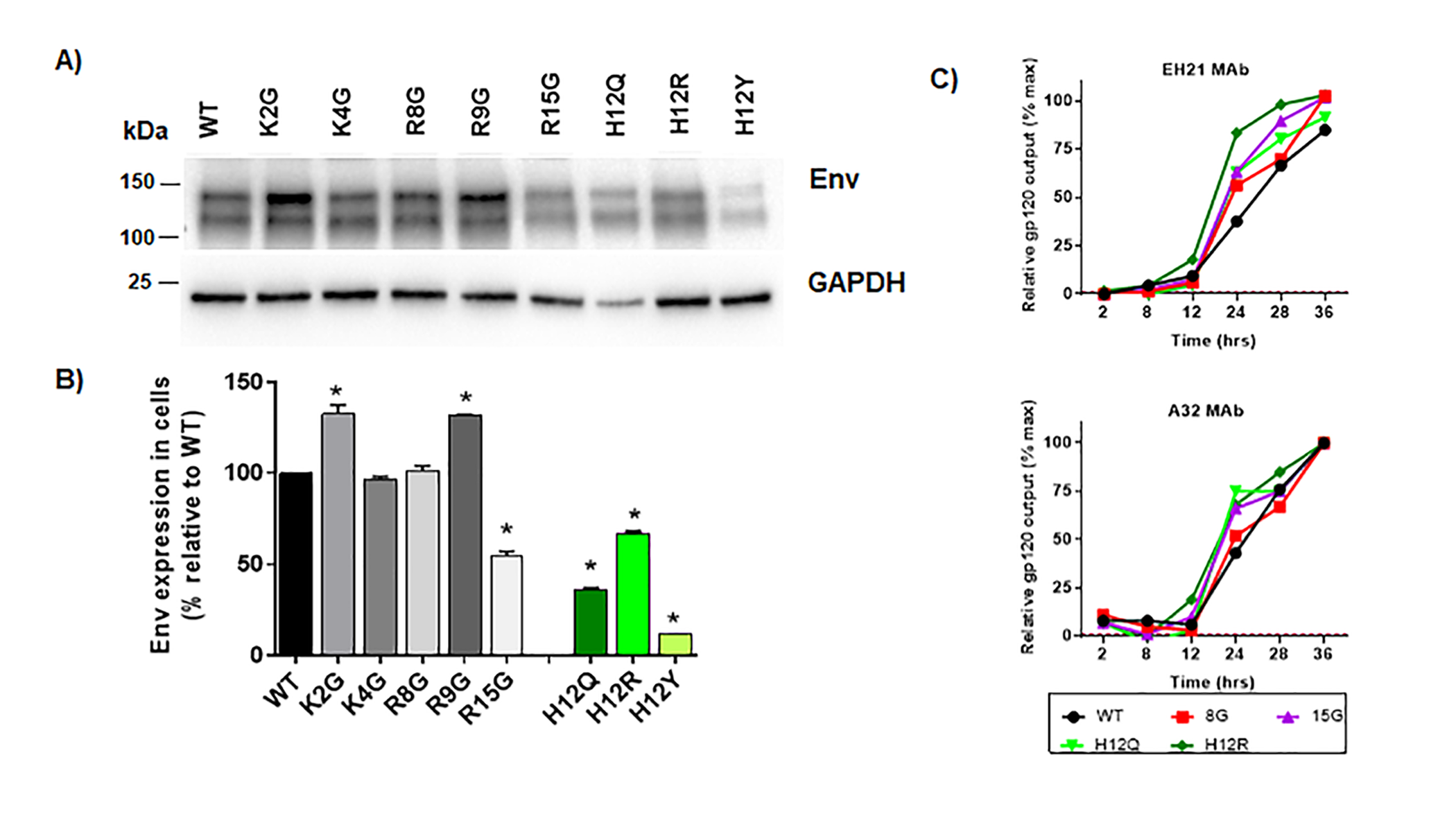

Supplement: S2 Fig — (A) Env expression in the cells as detected by an anti-gp120 MAb cocktail. WT and mutant REJO constructs were transfected into 293T cells. The cells were washed with PBS, lysed, and analyzed by Western blot. An anti-gp120 MAb cocktail (V3: 391/95-D, 694/98-D, 2219, 2558; C2: 847-D, 1006-30D; C5: 450-D, 670-D) was used as a probe. GADPH was used as loading control. (B) Expression of mutant Env in the cells relative to WT (100%).*, p< 0.01 (ANOVA). (C) Rate of Env production in cells as measured by MAb EH21 specific for a linear C1 epitope and MAb A32 specific for a conformation-dependent epitope involving C1, C2 and C4. 293T cells were transfected by REJO WT or mutant plasmids and harvested from 2 to 36 hrs after transfection. Env was captured onto ELISA plate by anti-C5 polyclonal sheep antibody and reacted with MAbs EH21 or A32. (TIF) [file ppat.1006812.s005.tif]

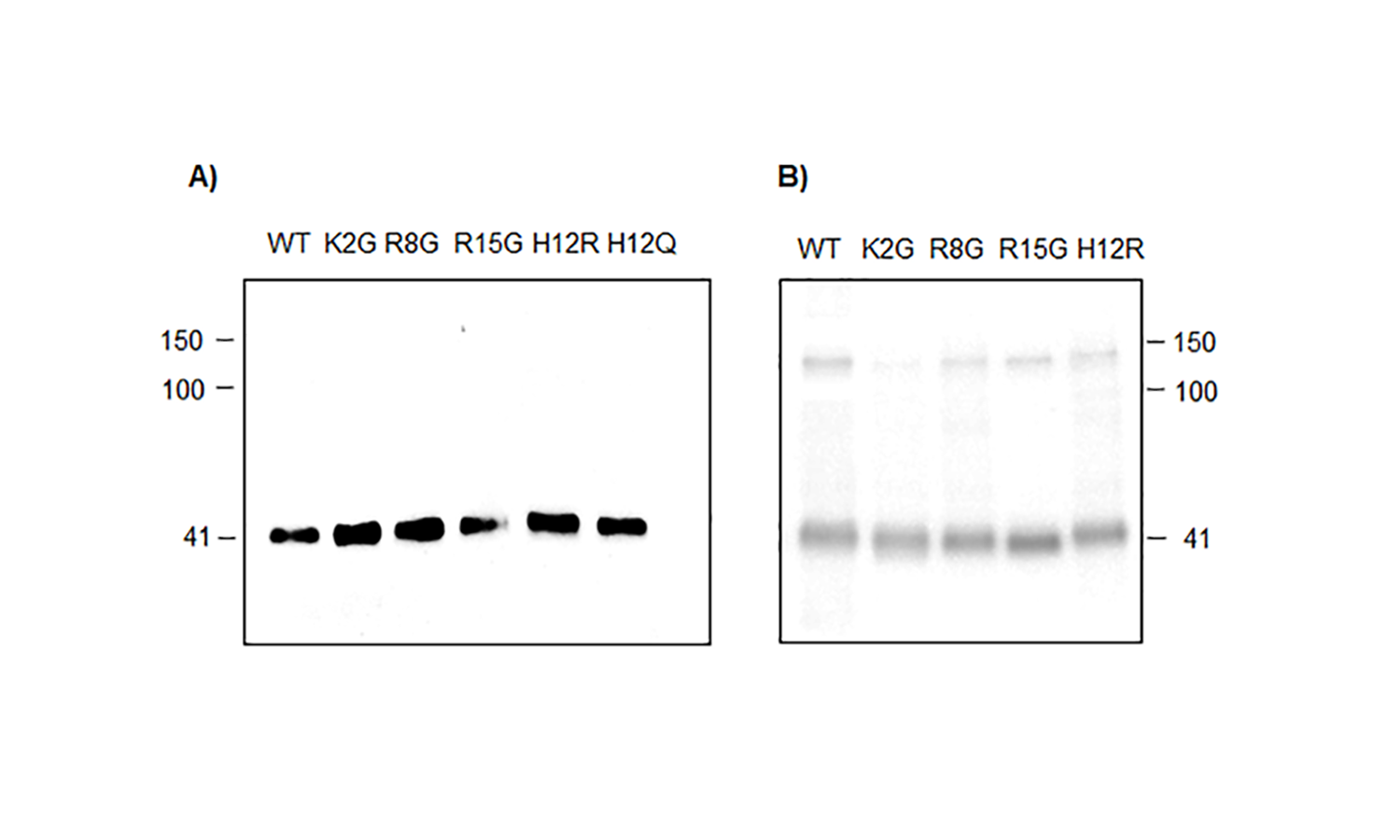

Supplement: S3 Fig — Western blots prepared as in Fig 6C were probed with A) anti-gp41 MAb cocktail (181-D, 240-D, 246-D, 167–7, 1367, 2295, 2556; 1μg/ml each), or B) gp41 MPER-specific MAb 2F5 (2 μg/ml). (TIF) [file ppat.1006812.s006.tif]

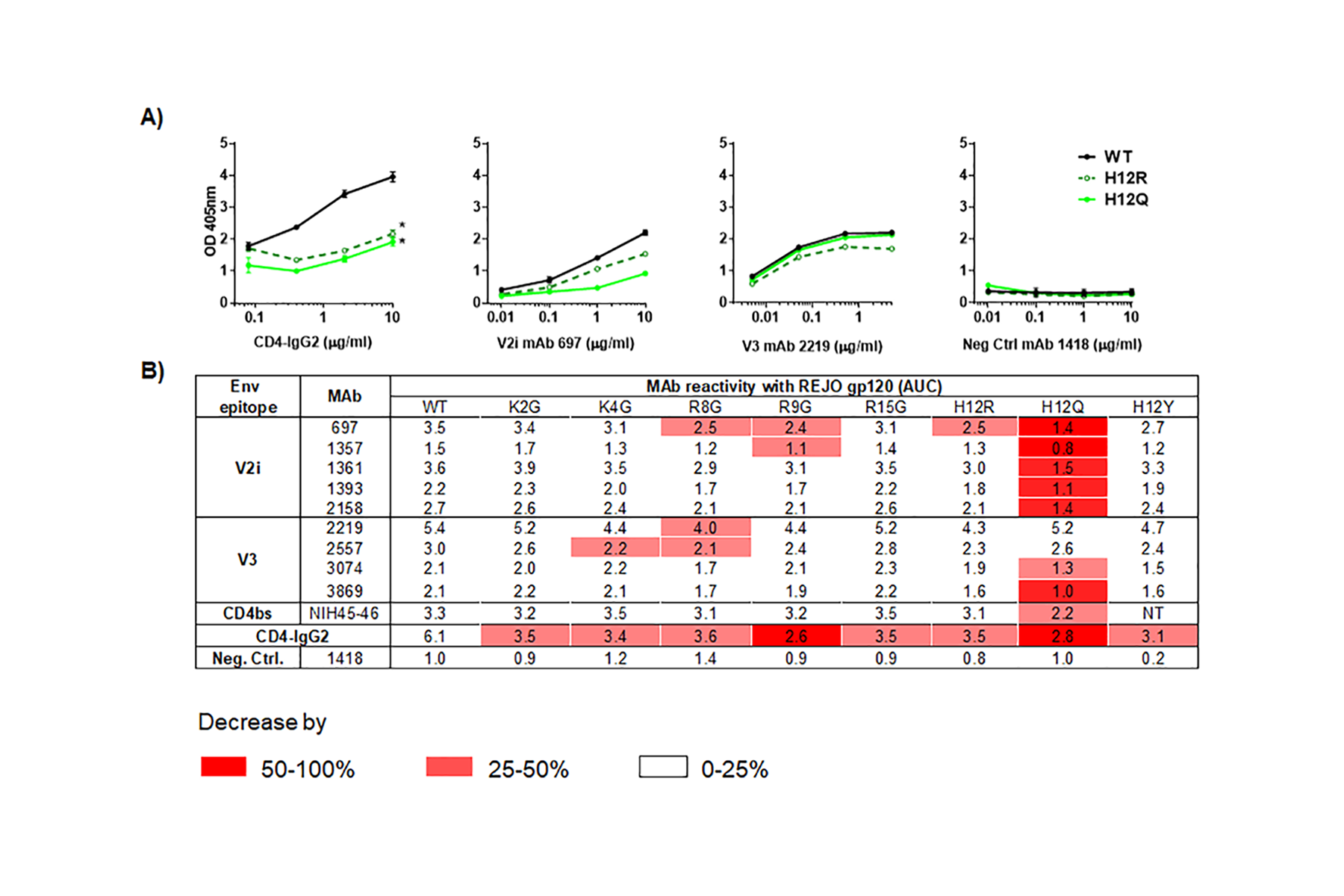

Supplement: S4 Fig — Virus lysates in 1% Triton-X100 containing equivalent constant amounts of gp120 from WT and mutant viruses were added to ELISA wells coated with sheep anti-C terminal gp120 antibodies and the captured gp120 proteins were reacted with MAbs or CD4-IgG2. The MAbs were titrated ten-fold from 10 μg/ml, while CD4-IgG2 was titrated five-fold from 10 μg/ml. A) Titration curves from representative MAb-virus pairs were shown. B) AUC values were calculated from all titration curves and the decreased levels of MAb binding to mutant gp120 versus WT were color-coded. (TIF) [file ppat.1006812.s007.tif]

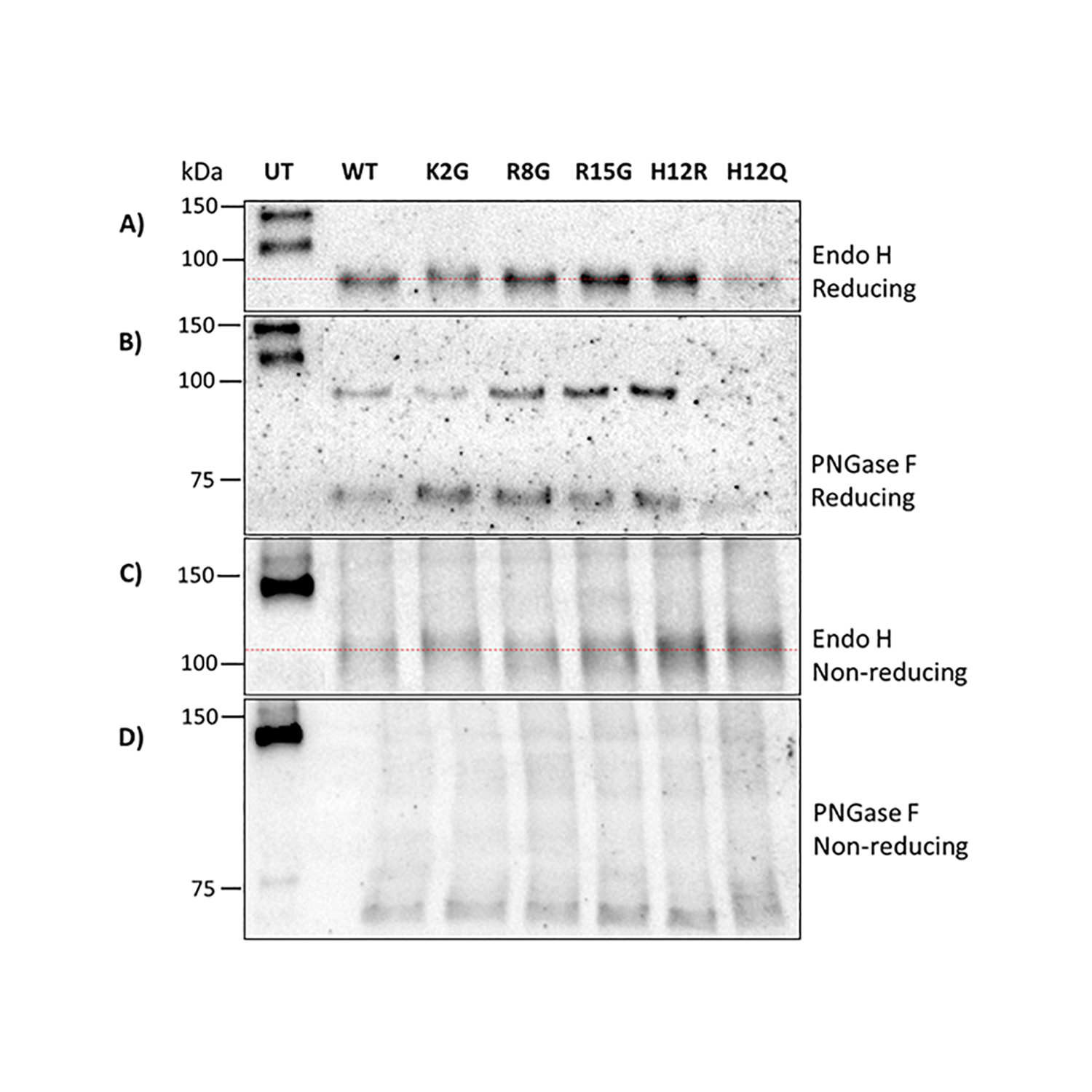

Supplement: S5 Fig — Sucrose-pelleted REJO WT and mutant virions were treated by Endo H or PNGase F under reducing (A and B respectively) or native non-reducing conditions (C and D respectively). All samples were then run on SDS-PAGE (10%) under reducing condition, and the blots were probed with anti-gp120 MAb cocktail. Untreated REJO WT (UT) was included for comparison. Red dotted lines are shown to highlight the observed changes in mobility shift of WT vs mutant Env proteins. (TIF) [file ppat.1006812.s008.tif]

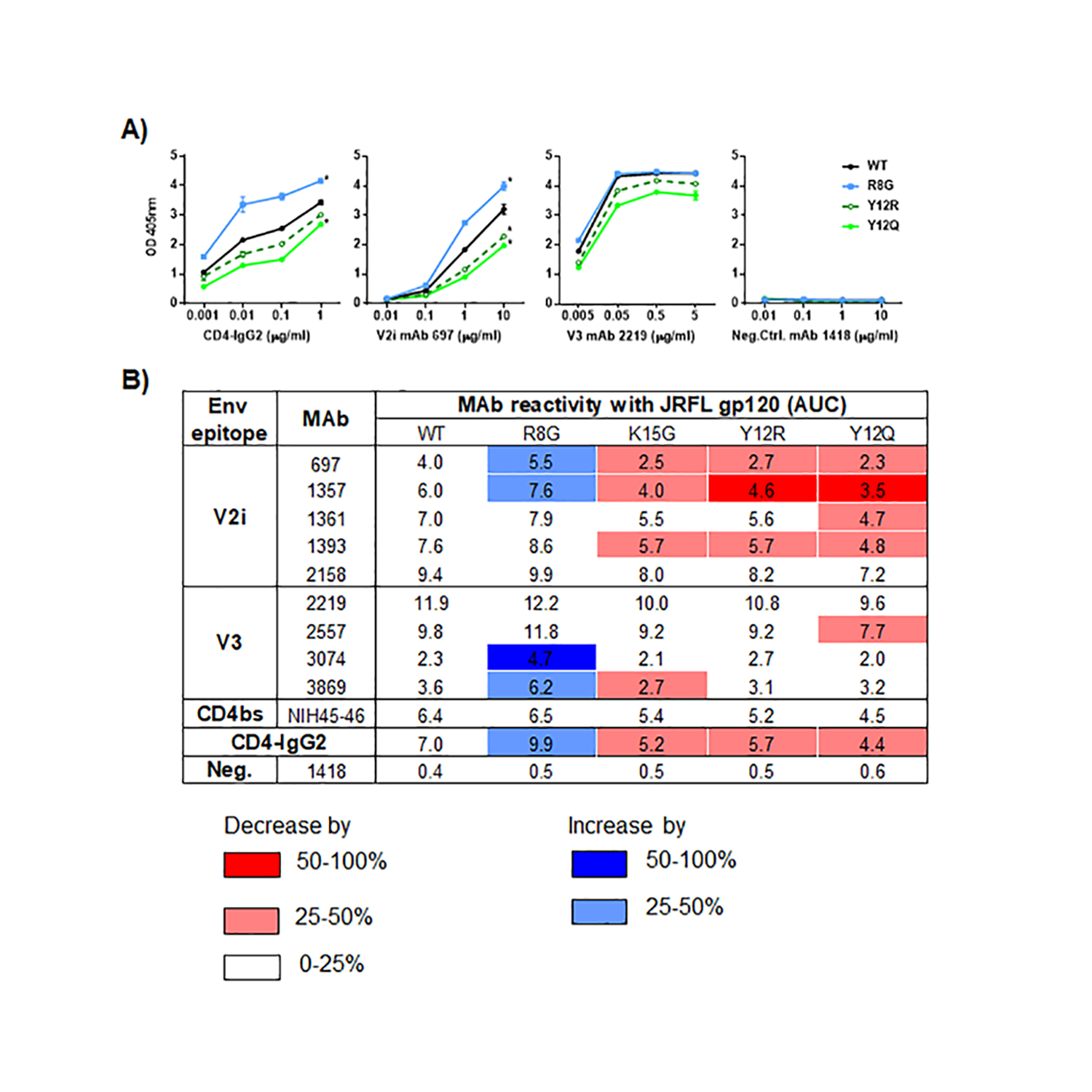

Supplement: S6 Fig — (A) Titration curves from representative MAb-virus pairs showing reactivity of gp120 from JRFL WT and SP mutant viruses with MAbs to V2i, V3, and the CD4bs and with CD4-IgG2. Virus lysates in 1% Triton-X100 containing equivalent constant amounts of gp120 from WT and mutant viruses were added to ELISA wells coated with sheep anti-C terminal gp120 antibodies and the captured gp120 proteins were reacted with MAbs or CD4-IgG2. The MAbs were titrated ten-fold from 10 μg/ml. B) AUC values were calculated from all titration curves and color-coded to show increased or decreased levels of MAb binding to mutant gp120 versus WT. (TIF) [file ppat.1006812.s009.tif]

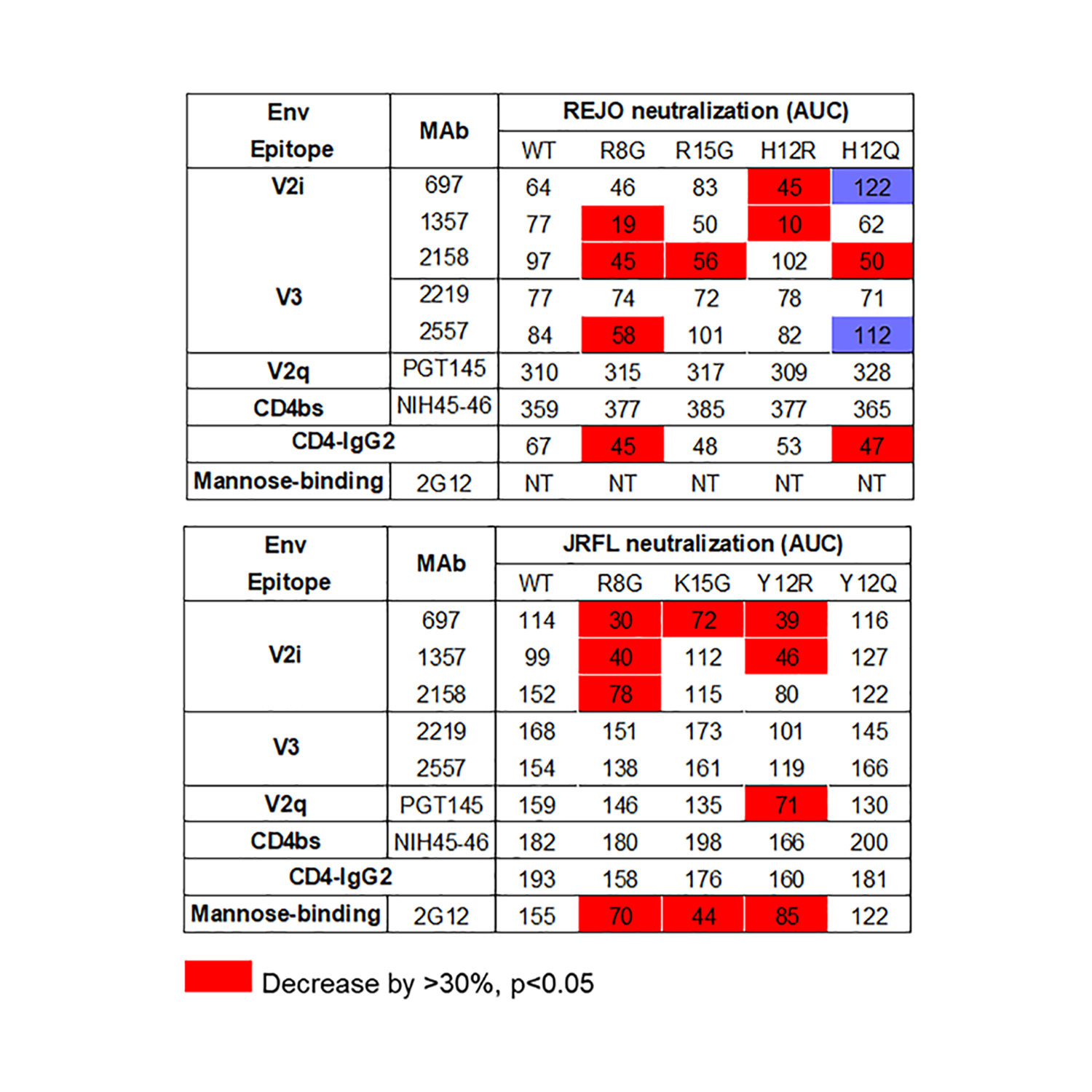

Supplement: S7 Fig — AUC values from Figs 3 and 10 are presented together to show the comparable effects of analogous SP mutations on neutralization of REJO and JRFL. (TIF) [file ppat.1006812.s010.tif]
